# Calcium Biofortification: Three Pronged Molecular Approaches for Dissecting Complex Trait of Calcium Nutrition in Finger Millet (*Eleusine coracana*) for Devising Strategies of Enrichment of Food Crops

**DOI:** 10.3389/fpls.2016.02028

**Published:** 2017-01-17

**Authors:** Divya Sharma, Gautam Jamra, Uma M. Singh, Salej Sood, Anil Kumar

**Affiliations:** ^1^Department of Molecular Biology and Genetic Engineering, College of Basic Sciences and Humanities, Govind Ballabh Pant University of Agriculture and TechnologyPantnagar, India; ^2^International Rice Research Institute Division, International Crops Research Institute for the Semi-Arid TropicsPatancheru, India; ^3^Indian Council of Agricultural Research-Vivekananda Institute of Hill AgricultureAlmora, India

**Keywords:** calcium, biofortification, bioavailability, finger millet, molecular breeding, functional genomics, transgenics

## Abstract

Calcium is an essential macronutrient for plants and animals and plays an indispensable role in structure and signaling. Low dietary intake of calcium in humans has been epidemiologically linked to various diseases which can have serious health consequences over time. Major staple food-grains are poor source of calcium, however, finger millet [*Eleusine coracana* (L.) Gaertn.], an orphan crop has an immense potential as a nutritional security crop due to its exceptionally high calcium content. Understanding the existing genetic variation as well as molecular mechanisms underlying the uptake, transport, accumulation of calcium ions (Ca^2+^) in grains is of utmost importance for development of calcium bio-fortified crops. In this review, we have discussed molecular mechanisms involved in calcium accumulation and transport thoroughly, emphasized the role of molecular breeding, functional genomics and transgenic approaches to understand the intricate mechanism of calcium nutrition in finger millet. The objective is to provide a comprehensive up to date account of molecular mechanisms regulating calcium nutrition and highlight the significance of bio-fortification through identification of potential candidate genes and regulatory elements from finger millet to alleviate calcium malnutrition. Hence, finger millet could be used as a model system for explaining the mechanism of elevated calcium (Ca^2+^) accumulation in its grains and could pave way for development of nutraceuticals or designer crops.

Mineral malnutrition is affecting one half of the world’s population. People have no access to a variety of minerals such as iron, calcium, zinc, magnesium, and copper. Most of the staple food crops such as rice, wheat and maize which constitute the major part of the diet of people are often deficient in these macro/micronutrients, thus insufficient to meet the daily needs (Hirschi, 2009). Deficiency of these minerals leads to an increasing risk of diseases such as rickets, osteoporosis, anemia, hypertension etc. It has been estimated by the Food and Agriculture Organization (FAO) that the world’s population will reach 9.1 billion by 2050 (Food and Agriculture Organization [FAO] of the United Nations, 2009). Hence, to meet the food demands of such a large population the quantity as well as the quality of food needs to be improved in terms of their nutritional value.

Calcium plays a very important role in the development of bones and teeth. Besides, it is required for a number of basic regulatory functions such as: contraction and relaxation of muscles, transmission of nerve impulses, coagulation of blood, activation of enzymatic reactions, stimulation of hormonal secretion and many other processes including synaptic plasticity, cell proliferation and cell death (Pravina et al., 2013). Calcium deficiency is a serious health problem which prevails in developed as well as in the developing world. Insufficient intake of calcium in human diets has been linked to diseases such as rickets (Heaney, 1993; Chan et al., 2007) and osteoporosis (Bhatia, 2008; Pettifor, 2008), both of which are the underlying cause for low bone density and poor bone growth. Eighty percent of the people who suffer from osteoporosis are women. Intake of calcium (Ca^2+^) in pregnant women is predominantly important for fetal skeleton development, increasing birth weight prenatal hypertension and avoidance of pre-clamsia (Chan et al., 2006). The human dietary intake of calcium in the majority of cases is below the recommended daily intake (RDI) of 800–1,300 mg per capita, and this is believed to result in widespread health and economic costs (Kranz et al., 2007).

Various approaches used to improve the calcium (Ca^2+^) content in grains/edible portion of crop plants include conventional breeding methodologies, marker assisted selection, transgenic technology and biofortification. Conventional plant breeding which aims in improving the target traits mainly by introgression from donor lines through hybridization has number of drawbacks such as the time required for breeding of cultivars, linkage drag, and absence of sufficient genetic variability in the primary gene pool with respect to a particular trait (Collard and Mackill, 2008). Molecular markers are considered best for indirect selection of traits and to avoid linkage drag. The association of markers with genes/QTLs controlling the traits of economic importance has been used for indirect marker assisted selection. However, genetic markers spread across the whole genome allow not only identification of individual genes associated with complex traits by QTL analysis but also the exploration of genetic diversity with regard to natural variation and can be extended for identification of major genes for nutritional traits. The genome wide markers saturating the whole genome are being used for genomic selection and results are encouraging in maize, wheat and barley (Piepho, 2009; Crossa et al., 2010; Guo et al., 2010; Heffner et al., 2011; Zhao et al., 2014). Another approach used for calcium biofortification is transgenics, by manipulating Ca^2+^ transporter genes. Several attempts have been made to increase calcium content in plant tissues and up to 300% increase of calcium content is recorded in plants (Park et al., 2005a; Kumar et al., 2014).

Hence, molecular marker assisted selection and transgenic technology are the two widely used approaches for biofortification to overcome the problem of “Hidden hunger,” which is the dietary insufficiency of one or more micronutrients such as calcium. However, for successful accomplishment of biofortification, high throughput ‘omics’ technologies provide useful insights and opportunities for development of third generation molecular markers, and identification of candidate genes for development of transgenics for ensuring nutritional security. The techniques of functional genomics along with the help of bioinformatics tools help in isolation and characterization of genes of nutritional importance. Genomics has also given us the information of complete genomes of a large number of food plants (Varshney et al., 2006). One of the main pillars of genomic studies is the development of high-throughput DNA sequencing technologies, collectively known as Next Generation Sequencing (NGS) methods, which can readily provide Single Nucleotide Polymorphism (SNPs) information of important nutrient rich genes (Govindaraj et al., 2015). Transcriptome sequencing gives information about the functional genes expressed in specific tissues of an organism at any given time in response to nutritional changes or other changes in the growth conditions. Transcriptome data is highly useful not only to know the gene content and transcriptional status in various tissues but also helps in identifying SSRs and SNPs in the genic regions, which can be converted to gene-based markers (Narina et al., 2011). Further, proteomics has helped researchers understand the effects of proteins on plant mineral homeostasis. It helps in monitoring the changes in proteins under different developmental and environmental conditions, as guided by the genome and signaled by the transcriptome.

This review comprehensively describes the three pronged molecular approaches for augmenting grain calcium content in finger millet: Molecular breeding, Functional Genomics and Transgenics. It also explains the challenges faced in a successful biofortification program and strategies for biofortification of calcium in plants for improved nutrition and development of functional foods. Further, it describes the mechanism of calcium uptake and transport from source to sink organs in plants and the molecular players involved in it.

## Biofortification: Major Challenges And Issues

Biofortification is the development of nutrient-dense staple crops using the best conventional breeding practices and modern biotechnology, without sacrificing agronomic performance and important consumer-preferred traits (Nestel et al., 2006). Plants are the ultimate source of nutrients in human diet. However, majority of the essential vitamins and minerals are lacking in all our staple food crops. Although, a balanced diet provides sufficient nutrients but most of the human population, particularly in developing countries depends upon staple cereals, such as rice or maize, which fail to provide the full complement of essential nutrients. Malnutrition has become a significant public health issue in most of the developing world (Müller and Krawinkel, 2005). One way to tackle this problem is through the enrichment of staple crops to increase their essential nutrient content. Several different tactics for biofortification have been adopted including addition of the appropriate mineral as an organic compound to the fertilizer, improving the nutritional content of plants by conventional breeding in combination with mutagenesis and the use of marker- assisted selection to introgress such traits into widely cultivated, adapted genotypes. Although breeding-based strategies for biofortification are unproven as yet, they have the potential to become sustainable, cost effective and reach remote rural populations (Bouis, 2003; Genc et al., 2005). It is argued that once mineral-dense lines have been developed, there will be little additional cost in incorporating them into on-going breeding programs (Welch and Graham, 2002; Bouis, 2003; Timmer, 2003), and it has been reported that seed of mineral dense crops produce more vigorous seedlings on infertile soils (Rengel and Graham, 1995). To implement successful biofortification programs through plant breeding there is need for a comprehensive exploration of potential genetic resources in the form of land races, wild species and an in-depth understanding of the physiological and genetic basis of mineral nutrients accumulation in staple food crop. It therefore becomes necessary to understand the genes and processes involved in grain mineral accumulation in order to couple the information with marker/genomics assisted selection, for efficient enhancement of grain mineral content.

A breeding program aiming at development of new genotypes with high Ca^2+^ concentration first requires existence of useful genetic variation for Ca^2+^ accumulation in grain. Little information is, however, available about the genetic control and molecular physiological mechanisms contributing to high accumulation of Ca^2+^ and other micronutrients in grain of different genetic materials.

## Bioavailability: A Complex Determining Factor

Plant foods contain substances (i.e., antinutrients) that interfere with the absorption or utilization of these nutrients in humans (Welch and Graham, 1999). Thus, efforts should be made toward increasing the concentrations of “promoter substances” (stimulating the absorption of essential mineral elements) and reducing the concentrations of “antinutrients” (interfering with their absorption) of the biofortified crops (White and Broadley, 2005). The best described promoter substances are certain vitamins, inulins and cysteine amino acid. Vitamin E, D, choline, niacin and provitamin A, help in the absorption of Se, Ca, P, Fe, and Zn (Carvalho and Vasconcelos, 2013). Finger millet contains both water soluble and liposoluble vitamins: thiamin, riboflavin, niacin, and tocopherols (Obilana and Manyasa, 2002), which could act as potential promoter substances for crop biofortification. Utilization of the maximum nutrient potential of the millets is limited by the presence of phytates, phenols, tannins and enzyme inhibitors. Among millets, finger millet has been reported to contain high amounts of tannins ranging from 0.04 to 3.74% of catechin equivalents (Rao et al., 1994; Antony and Chandra, 1999). Phytate content in finger millet as observed by various authors has been found to be in range 0.679–0.693 g/100 mg (Antony and Chandra, 1999). It is the main phosphorous store in mature seeds, has a strong binding capacity and readily forms complexes with multivalent cations and proteins (Haug and Lantzsch, 1983). Finger millet has been found to contain 41% phytic phosphorus as percentage of total phosphorus (Deosthale, 2002). The dietary phytic acid binds not only with the seed derived minerals but also with other endogenous minerals encountered in the digestive tract (Raboy, 2000). Another group of anti-nutritional compound is polyphenols, which contains more than one phenol unit or building block per molecule (Carvalho and Vasconcelos, 2013). The level of polyphenols in cereal seeds can be reduced by incubation with polyphenol oxidase which, when combined with a phytase-mediated phytate reduction, shows significant increase in the availability of iron (Matuschek et al., 2001). On an average, finger millet genotypes contain 0.04–3.47% polyphenols (Chethan and Malleshi, 2007). Rao and Muralikrishna (2002) found proto-catechuic acid (45.0 mg/100 g) as the major free phenolic acid in finger millet grains. Among bound phenloic acids, ferulic and *p*-coumaric acid are the major fractions and account for 64–96 and 50–99% of total ferulic and *p*-coumaric acid content of finger millet grains, respectively, (Devi et al., 2014).

**FIGURE 1:**
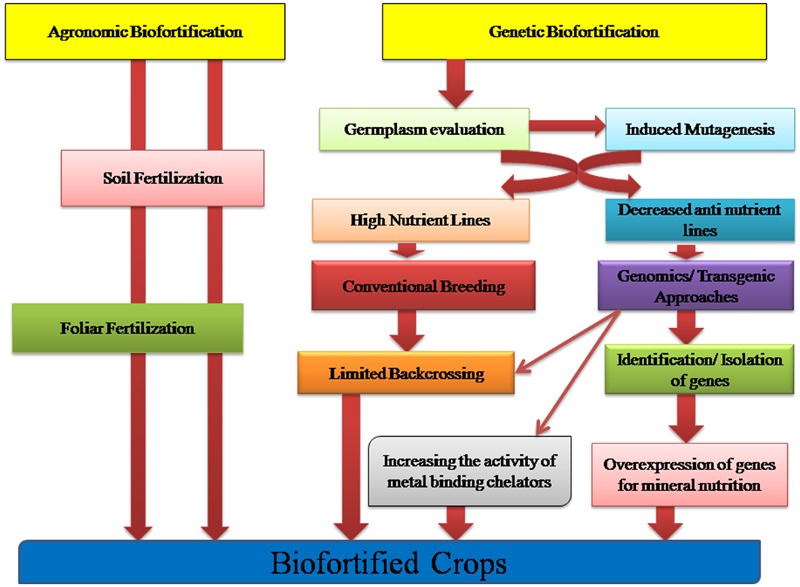
**Crop Biofortification approaches: Agronomic and Genetic Biofortification strategies aiming to increase the bioavailability and accumulation of micronutrients in the edible tissues of crops**.

Numerous complexities pervade the determination of bioavailability of micronutrients in plant foods to humans. Determining the bioavailability of a particular micronutrient to an individual eating a mixed diet in a given environment is actually governed by the interaction of a multitude of factors (Fairweather-Tait and Hurrell, 1996; House, 1999; Van Campen and Glahn, 1999; Graham et al., 2001). Two approaches of conventional and genetic biofortification as given in **Figure 1** are being currently practiced. Through plant breeding approaches one could select genotypes with low concentration of anti-nutrients or alternatively molecular biologists alter genes in staple crops so as to reduce or completely eliminate these anti-nutrients. However, doing so is associated with many risk factors and should be done with utmost care because many of the anti-nutrients are major plant metabolites that might play important roles in plant abiotic stress resistance, plant metabolism and in resistance of plant to crop pests or pathogens (Graham et al., 2001). Moreover, some of the anti-nutrients such as phytate and polyphenols might play key beneficial roles in human diets by acting as anti-carcinogens or by promoting health in other ways such as decreasing the risk of heart diseases or diabetes (Zhou and Erdman, 1995; Anonymous, 1996; Saied and Shamsuddin, 1998; Shamsuddin, 1999). Therefore, molecular biologists and plant breeders must consider the possible negative consequences of altering the concentration of these anti-nutrients in staple food crops.

## Plants: The Power House of Calcium

It’s common knowledge that we need calcium for strong bones, teeth and besides this for a number of regulatory functions in our body. But, the best source of dietary and supplemental calcium is not understood yet. Following dairy products, plant products prove to be the largest potential contributor to Ca^2+^ intake (Weaver and Plawecki, 1994; Weaver et al., 1999; Lanham-New, 2008). Ever since the beginning of mankind, plant based foods constitute one of the potential nutrient sources in human diet (Carvalho and Vasconcelos, 2013). For proper calcium (Ca^2+^) absorption we need to consume food sources that contain types of calcium that are easily digested, assimilated and absorbed. Because of the calcium-magnesium ratio in dairy products, our body is not capable of absorbing the calcium (Bennett and Sammartano, 2012). However, calcium is naturally bound with other minerals, vitamins, proteins, and phyto-nutrients in the plants. Plant foods provide our body with calcium which is safe to absorb and directs our body to store it in our bones. Most of the staple foods, however, (e.g., Rice, wheat, and maize) have very low amount of calcium (Jeong and Guerinot, 2008). Plant based calcium sources includes green leafy vegetables, nuts, fruits, seeds, sea vegetables which help us to gain more calcium.

To implement a successful biofortification program for increasing calcium content in plants, comprehensive exploration of potential genetic resources in the form of land races and wild species is required. Also there is a need to have an in depth knowledge of physiological and genetic basis of calcium accumulation in staple food crops. It is therefore necessary to understand the genes and processes involved in grain calcium accumulation so as to blend this information with marker assisted selection for enhancing grain calcium concentration.

### Finger Millet: A High Calcium Accumulating Crop

Finger millet is a potential staple crop cultivated mostly in Eastern and Central Africa and India. It ranks fourth in importance among millets in the world after sorghum, pearl millet, foxtail millet and commonly referred as *ragi, mandua*, bird’s foot millet, caracan millet and African millet (Upadhyaya et al., 2007). Nutritionally, finger millet is an excellent sources of nutrients especially calcium, other minerals and dietary fiber. The mineral composition of finger millet grains is highly variable. The mineral content of food grains is affected by the presence of genetic factors and environmental conditions prevailing in particular growing region (Singh and Raghuvanshi, 2012; Singh et al., 2014). Finger millet contains a fair amount of protein (7.3%) (Malleshi and Klopfenstein, 1998), dietary fiber (15–20%) (Chethan and Malleshi, 2007), and a rich source of calcium (344 mg/ 100 g) (Gopalan et al., 1999; Bhatt et al., 2003) and iron (3.7–6.8 mg/ 100 g) (Barbeau and Hilu, 1993). Ravindran (1991) estimated the protein content of finger millet to be 9.8%, that of calcium, oxalate and phytic acid to be 0.24, 0.44, and 0.48%, respectively. In several studies on estimation of calcium content in different genotypes of finger millet, high calcium values have been reported. Calcium content varied from 162 to 487 mg/100 g with a mean value of 320.8 mg/100 g grain in 36 genotypes of finger millet (Vadivoo et al., 1998), 293–390 mg/100 g in six varieties of finger millet (Babu et al., 1987); 50–300 mg/100 g in another set of six varieties (Admassu et al., 2009). Furthermore, very high calcium content, 450 mg/100 g (Panwar et al., 2010) and 489 mg/100 g (Upadhyaya et al., 2011) has been reported in few finger millet genotypes. It has also been found that the average calcium content (329 mg/100 g grain) in white genotypes was considerably higher than the brown (296 mg/100 grain) genotypes (Seetharam, 2001). Besides high nutritional value, finger millet has high grain yield potential in the range of 4-5 tons/ha (Food and Agriculture Organization [FAO] of the United Nations, 2008). Thus, finger millet is up-coming as an important food crop due to its exceptionally high calcium (Ca^2+^) content. It is not only an excellent source of dietary calcium (Ca^2+^) but also an excellent model to explore the genetic control and molecular mechanisms contributing to high grain calcium (Ca^2+^) content.

The millet is also well known for its health benefits such as hypocholesterolemic, hypoglycaemic and anti-ulcerative properties (Chethan and Malleshi, 2007). Further, the crop is productive in wide range of environments and growing conditions from Karnataka in South India to foothills and middle hills of Himalayas in North India and Nepal, and throughout the middle-elevation areas of Eastern and Southern Africa (Kumar et al., 2016). Finger millet being a promising source of micronutrients and protein (Malleshi and Klopfenstein, 1998), can play a significant role in alleviation of micronutrient and protein malnutrition, which affects more than one half of the world’s population, especially women and preschool children in most countries of South-east Asia and Africa (Underwood, 2000).

## Molecular Players Involved in Calcium Transport

### Source to Sink Transport in Root, Stem, Leaves and Developing Spikes

Calcium (Ca^2+^) is normally acquired by root from the soil solution in free ionic form. The outer layers of root, the epidermal cells and their elongated projection (root hairs) get in touch with the soil solution where Ca^2+^ are present in milli-molar range. Through a variety of Ca^2+^ permeable channels, which includes cyclic nucleotide gated channels (CNGCs), glutamate receptor like (GLR) proteins, two-pore channels (TPCs) and mechano-sensitive Ca-permeable channels (MSCCs) calcium (Ca^2+^) enters inside the root epidermal cells, where it moves both apoplastically and symplastically to the cortex and then to the stele. Further, it may reach xylem either apoplastically through extracellular spaces or symplastically, by entering root cells and moving from cell to cell through plasmodesmata.

When the root is young, metaxylem and protoxylem are the main routes of Ca^2+^ transport while in later stage when the vessels are fully conductive, central xylem becomes the major sink. After being released from root, Ca^2+^ are transported in the apoplast and xylem vessels of the shoot where the cation exchange capacity (CEC) of xylem walls and intensity of transpiration is the major factor for Ca^2+^ transportation (McLaughlin and Wimmer, 1999). Ca^2+^ present in the xylem sap exchanges with bound Ca^2+^ from the xylem walls; the movement of an individual Ca^2+^ will be in a series of jumps between exchange sites (Atkinson et al., 1992). Trunk xylem tissues deliver Ca^2+^ inside the leaf tissue via vein extensions. The calcium (Ca^2+^) delivery and distribution in leaves is mostly dependent on the pathway of water flow to and through the leaves (Gilliham et al., 2011). After separation of mineral cations from water, Ca^2+^ moves slower in apoplast. This could be due to the fact that Ca^2+^ transport within the leaf is not only by mass flow but also via an extracellular pathway (Canny, 1993). Growing part of leaf tissue requires high Ca^2+^ concentration, hence providing a sink for Ca^2+^ movement.

Members of calcium signaling and transport genes are reported to be involved in calcium uptake, transport and accumulation at cellular levels in plants. These include various types of calcium sensors viz., calcium dependent protein kinases (CDPKs), Calcinurin- B like protein kinases (CIPKs), calmodulin dependent protein kinases (CaMKs), calcium/calmodulin dependent protein kinases (CCaMKs) and transporters genes viz., Ca^2+^ -ATPases, Calcium/cation exchangers and calcium channels (Singh et al., 2015). Calcium transporters are actively involved in the uptake and transport of calcium in the cells, while calcium sensors are involved in the regulation of these transporters (**Table 1**). Understanding the role of these genes and the availability of the calcium in rhizosphere might help in development of calcium biofortified plants. Mirza et al. (2014) analyzed the expression of calcium transporter and their regulator genes in different tissues at different growth stages in two finger millet genotypes differing in calcium content and reported higher expression of *CAX1* gene in roots of high calcium genotype GPHCPB45 in addition to two more genes (*TPC1* and *ATPase*) in most of the tissues at vegetative growth and developing spikes except flag leaf. Higher expression of *CAX1* in roots suggests that calcium uptake is taking place at high rate probably under the regulation of calmodulin independent pathway as the expression of calmodulin is invariably low in all vegetative tissues (Mirza et al., 2014). The expression of *CAX1* was observed to be low in root tissue as compared to those in other tissues as reported earlier (Carter et al., 2004; Cocozza et al., 2008; Conn and Gilliham, 2010). This suggests that although calcium uptake is taking place at high rate, it is not getting stored in the vacuole rather it is eﬄuxed in the root apoplast and is trafficked to the cells by means of the water transpiration stream. The calcium content in the leaves was higher than those in the root and stem tissue with higher content in GPHCPB45 leaves as compared to GPHCPB1. This indicates that higher transpiration might attribute to higher calcium (Ca^2+^) accumulation in GPHCPB45 genotype. Interestingly, the expression pattern of 14-3-3 gene was observed to be similar to *CAX1* gene in leaf tissue indicating that 14-3-3 might interact with the *CAX1* for calcium content regulation in leaves. Further in the developing spike (S1 – S4 stage), all the transporters exhibited an increased expression with higher expression in GPHCPB45 and hence correspond to the higher calcium (Ca^2+^) content (Mirza et al., 2014). Higher expression of *TPC1* and *CAX1* genes in GPHCPB45 indicates higher uptake and accumulation of calcium (Ca^2+^) in comparison to GPHCPB1 in the developing spikes. All the regulatory proteins also exhibited expression patterns similar to the transporter genes in developing spike with generally a higher expression in GPHCPB45 genotype. *CaM* exhibited a similar expression pattern as Ca^2+^ *ATPase* gene indicating that this CaM isoform might be specific to developing spike and activating the Ca^2+^ ATPase through binding to the CBD domain (Amtmann and Blatt, 2009). *CAM1* was strongly expressed during developing spikes of high grain calcium genotype along with *CAX1*. Insilico analysis showed that EcCAM interacts with aquaporin indicating calcium is probably delivered to developing spikes via mass flow of water (Kumar et al., 2014). The results indicate that calmodulin independent and dependent pathways cause greater stimulation of transport machinery operative in vegetative and spike tissues leading to higher accumulation of calcium from source to sink. A *CAX*–*CaM* dual gene construct may be designed for targeted co-expression under grain endosperm cell-specific promoters to fortify cereals with bioavailable calcium. In flag leaf, the expression of all the transporter and regulatory genes was found to be down regulated, however, the calcium content in flag leaf was highest among all the tissues in both the genotypes. This indicates that the calcium accumulation in flag leaf is largely dependent on the transpirational pull and isoforms.

**Table 1:** List of Ca2+transporters in plants.

Name of transporters	Function	Domain	Location	Regulation	References	Remarks
**1) Ca^2+^ channels**						
**(A) Cyclic nucleotide gated channels**						
*Arabidopsis thaliana* (20 members)	Ca^2+^ transport	TM, cNMP, CBD	PM	cNMPs, Ca^2+^-CaM	Kaplan et al., 2007	Lower ion selectivity allowing a number of cations (K+, Na+, Cs+, Pb2+, Sr2+ etc) to cross plasma membrane
*Oryza sativa* 10 members	Ca^2+^ transport	TM, cNMP, CBD	PM	cNMPs, Ca^2+^-CaM	Ward et al., 2009	
*Poplar trichocarpa* (12 members)	Ca^2+^ transport	TM, cNMP, CBD	PM	cNMPs, Ca^2+^-CaM	Ward et al., 2009	
*Arabidopsis thaliana* (20 members)	Ca^2+^ Transport	TM, cNMP, CBD	PM	cNMPs, Ca^2+^-CaM	Kaplan et al., 2007	
**(B) Glutamate receptor homologs (GLRs)**						
*Arabidopsis thaliana* (20 members)	Ca^2+^ transport	TM, GBD	PM	Glutamate and glycine	Lacombe et al., 2001	Also permeable for Na^+^, K^+^ and Ba^2+^ ions.
*Oryza sativa* (13 members)	Ca^2+^ transport	TM, GBD	PM	Glutamate and glycine	Ward et al., 2009	
*Poplar trichocarpa* (61 members)	Ca^2+^ transport	TM, GBD	PM	Glutamate and glycine	Ward et al., 2009	
**(C) Two-pore channels**						
*Nicotiana tobacum* (NtTPC1a and NtTPC1b)	Ca^2+^ transport	TM, EF-hands	PM, TP	Sugar-induced depolarization and Ca^2+^	Kadota et al., 2004	Also permeable for Na^+^, K^+^ Ra^+^, Cs^+^, Mg2^+^ and Ba^2+^ ions.
*Oryza sativa* (OsTPC1)	Ca^2+^ transport	TM, EF-hand motif	PM, TP	Sugar-induced depolarization and Ca^2+^	Kurusu et al., 2004	
*Arabidopsis thaliana* (AtTPC1)	Ca^2+^ transport	TM, EF-hand motif	PM, TP	Sugar-induced depolarization and Ca^2+^	Furuichi et al., 2001; Islam et al., 2010	
Wheat (TaTPC1)	Ca^2+^ transport	TM, EF-hand motif	PM, TP	Sugar-induced depolarization and Ca^2+^	Wang et al., 2005	
*Nicotiana tobacum* (NtTPC1a and NtTPC1b)	Ca^2+^ transport	TM, EF-hands	PM, TP	Sugar-induced depolarization and Ca^2+^	Kadota et al., 2004	
*Oryza sativa* (OsTPC1)	Ca^2+^ transport	TM, EF-hand motif	PM, TP	Sugar-induced depolarization and Ca^2+^	Kurusu et al., 2004	
**(D) Mechanosensitive Ca^2+^-permeable channels (MSCCs)**						
*Arabidopsis thaliana* (4 members)	Ca^2+^ transport	PLAC8 domain	PM	Touch induced	Haswell et al., 2008; Yamanaka et al., 2010	MSL9 and 10 are more permeable for Cl^-^ than Ca^2+^
**2)Ca^2+^ ATPase**						
**(A) P-type Ca^2+^ ATPase /Endoplasmic reticulum-type Ca^2+^-ATPase (ECA)/ IIA Type**						
*Arabidopsis thaliana* (4 members)	Ca^2+^ transport	TM and ATPase	ER,TP, PM, GO, NU	Energy from hydrolysis of ATP	Baxter et al., 2003; Pittman, 2011	Beside Ca^2+^, also transport Mn^2+^
*Oryza sativa* (3 members)	Ca^2+^ transport	TM and ATPase	ER,TP, PM, GO, NU	Energy from hydrolysis of ATP	Goel et al., 2012	
**(B) P-type Ca^2+^ ATPase /Autoinhibited Ca^2+^-ATPase (ACA)/ IIB Type**						
*Arabidopsis thaliana* (10 members)	Ca^2+^ transport	TM, CMBD, autoinhibitory and ATPase	PM, TP, ER, PL, GO, MT	Ca^2+^-CaM	Baxter et al., 2003	–
*Oryza sativa* (11 members)	Ca^2+^ transport	TM, CMBD, autoinhibitory and ATPase	PM, TP, ER, PL, GO, MT	Ca^2+^-CaM	Baxter et al., 2003; Goel et al., 2012
**3) Ca^2+^/H^+^ Exchangers/ Ca^2+^-Antiporter**						
*Arabidopsis thaliana* (6 members)	Ca^2+^transport	TM, auto-inhibitor	PM, TP, (MT and NU?)	Proton-motive force	Pittman, 2011	
*Oryza sativa* (6 members)	Ca^2+^ transport	TM, auto-inhibitor	PM, TP, (MT and NU?)	Proton-motive force	Goel et al., 2011	

*TM, transmembrane; cNMP, cyclic nucleotide monophosphate; CBD, calcium binding domain, GBF, glutamate binding domain; CMBD, calmodulin binding domain; PM, plasma membrane; TP, tonoplast; GO, golgibody, PL, plastid; NU, nuclear membrane; ER, endoplasmic reticulum, MT, mitochondria.*

**FIGURE 2:**
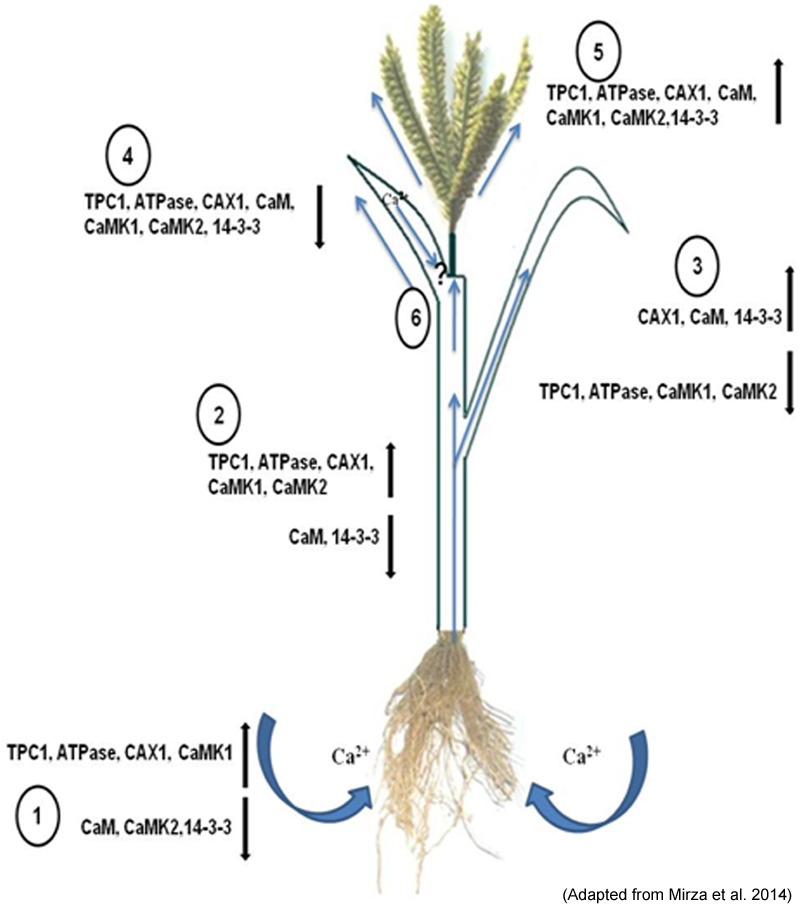
**Depiction of the role of the potential transporters and their regulatory genes during translocation of calcium (Ca^2+^) from the rhizosphere by the (1) root and root hair and translocation.** Along with the xylem stream through (2) stem, distribution to the (3) leaves and phloem loading for movement into the (4) flag leaf and (5) developing spike (6). The up (↑) and down (↓) regulation of these genes are given in the sketch diagram (Adapted from Mirza et al., 2014).

Briefly, the differential expression of genes shows differential spatial and temporal accumulation of calcium (Ca^2+^) in the two genotypes of finger millet. Studies on finger millet *CAX1* and *ATPase* might offer further insights in understanding their auto-inhibition and strategic regulation. However, higher transcript abundance does not always corroborate with higher gene product abundance and activity. There are many post translational modifications and other bottle necks that may alter the fate of transcriptional activity. The results of transcriptome needs further validation using knock in and knock out approaches. Thus, finger millet plant might as well befall as a model system for better understanding of the underlying genetic control and molecular physiological mechanisms contributing to high grain calcium. **Figure 2** depicts the role of potential transporters and regulatory genes involved in calcium (Ca^2+^) transport from source to sink in finger millet.

### Calcium Accumulation in Seed

Seed consists of filial tissues (aleurone and endosperm cell) and maternal tissues (seed coat and nucellar cell) with no direct vascular connection between them (Wolswinkel, 1992). Generally, differentiated vascular systems are restricted to seed tissues and are primarily comprised of phloem with no or limited xylem (Patrick, 1997). Phloem immobility of Ca^2+^ forces it to take different route for delivery in seed. The possibilities include the non-vascular symplastic pathway from funiculas, viz., xylem transfer cell, pigment strands, nucellar cell, or apoplastic (cell wall) routes (Van Bel, 1990). The calcium concentration in phloem-fed tissues, such as fruits, seeds and tubers is in general low in many crops (Karley and White, 2009), but finger millet seeds have been found to be accumulating very high calcium varying from 100 to 450 mg/100 g (Panwar et al., 2010). Therefore, it becomes an important target crop toward elucidating the genetic and epigenetic basis of calcium uptake, transport and accumulation in plant. The information generated through this could be utilized for biofortification of staple crops through transgenic approaches which relies on improving the uptake, translocation and accumulation in edible tissues or through adequate availability in their roots.

**FIGURE 3:**
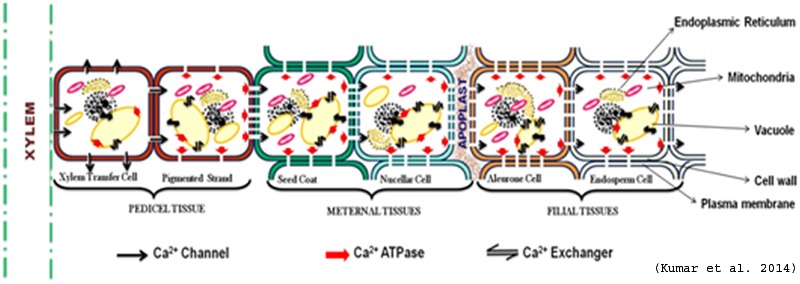
**Hypothetical model of Ca^2+^ transport in seed (Adapted from Kumar et al., 2014)**.

Based on the existing knowledge about calcium (Ca^2+^) transport and accumulation, a hypothetical pathway of seed calcium accumulation is proposed in **Figure 3**. In this model, it is proposed that Ca^2+^ from xylem passes through the pedicel tissues (Xylem transfer cell and pigmented strand) and then enters the maternal tissue (seed coat) with the help of transporters. It gets pumped toward the apoplast from seed coat, where it is absorbed by aleurone and then in endodermal cell. So, prior to uptake by the embryo, Ca^2+^ are deposited in the seed-coat apoplastic space abutting the surface of the embryo (Patrick, 1990). The seed coat is major Ca^2+^ storage tissue followed by aleurone layer and the endosperm (Nath et al., 2012). The contrasting distribution pattern of Ca^2+^ in different tissues of seed during seed development indicates the role of physiological and molecular mechanisms of their accumulation. The presence of prominent, insoluble calcium oxalate crystals (especially in seed coat) and calcium phytates in the embryo of mature seed has been reported to be an important factor for high grain calcium in plants (Barnabas and Arnott, 1990; Ilarslan et al., 2001).

Spatial distribution of calcium transporters within the cell provides the path for storage and movement of calcium (Ca^2+^). There is good evidence that Ca uptake from the apoplast is mediated by activity of Ca channels, which would be the most energetically efficient mechanisms because they utilize the large inward directed electrochemical potential gradient for Ca (Franceschi and Nakata, 2005). The occurrence of abundant Ca^2+^ channels in the cell membrane toward the xylem (funiculus) and Ca^2+^-ATPases toward endosperm might be responsible to pump Ca^2+^ in the seed (**Figure 2**). Once Ca^2+^ enters inside the seed it might get stored inside cell organelles *viz*., vacuole, ER etc. Vacuole is reported as the major reservoir of Ca^2+^ and up to 80% or more of the cellular volume of plant cells is occupied by vacuole (Pittman, 2011). The abundance of Ca^2+^ transporters especially antiporters on tonoplast contribute toward high calcium (Ca^2+^) in seeds (Conn et al., 2011; Punshon et al., 2012). Higher expression of Ca^2+^ transporters and calcium binding proteins (CaBPs) in the tonoplast membrane has been reported for the uptake and storage of Ca^2+^ in vacuole of leaf cell (Blumwald and Poole, 1986; Wang et al., 1995).

**FIGURE 4:**
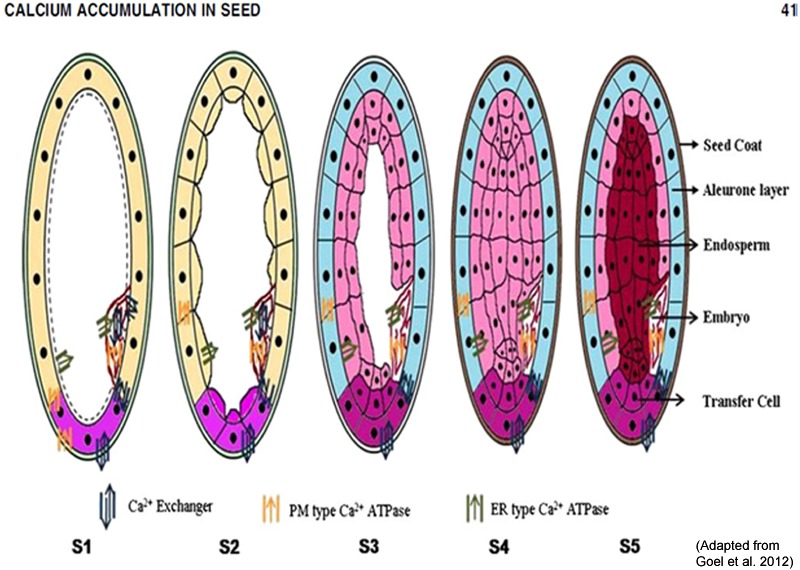
**A hypothetical model showing the possible mechanism of calcium accumulation in various stages of cereal grains development.** S1 (booting), S2 (spike emergence), S3 (pollination), S4 (Grain filling or milky) and S5 (seed maturation). (Adapted from Goel et al., 2012).

The mechanism of Ca transport inside seed is still not known, as Ca is phloem immobile and seed is fed mainly by phloem. Our preliminary study based on rice Ca sensor and transporter data suggests that Ca transporters (especially Ca Exchanger) are the main Ca transporting proteins that pump Ca inside seed. Based on the structural and expression analysis of the calcium transporter genes and available data in literature, a hypothetical model for accumulation of calcium in the cereal grains was proposed (Goel et al., 2011, 2012). The nine calcium transporter genes identified through the analysis of rice MPSS and microarray data were tested and one calcium ATPase and one calcium exchanger was found to be highly expressed in high seed calcium finger millet genotype. In finger millet, out of the five genes encoding calcium transporters and sensors (*Ca ATPase-01, CAX-01, TPC-01, CaMK-02* and one gene of 14.3.3) isolated from finger millet, *CAX1* showed strong expression in the later stages of spike development indicating its role in accumulating high amounts of calcium in seeds (Mirza et al., 2014). Therefore, it can be speculated that the same mechanism of grain calcium accumulation exists in finger millet as is found in rice. However, the model is probabilistic and experimental evidence like gain or loss of function studies are required to validate this model. **Figure 4** depicts a hypothetical model showing the possible mechanism of calcium accumulation in various stages of cereal grains development.

**FIGURE 5:**
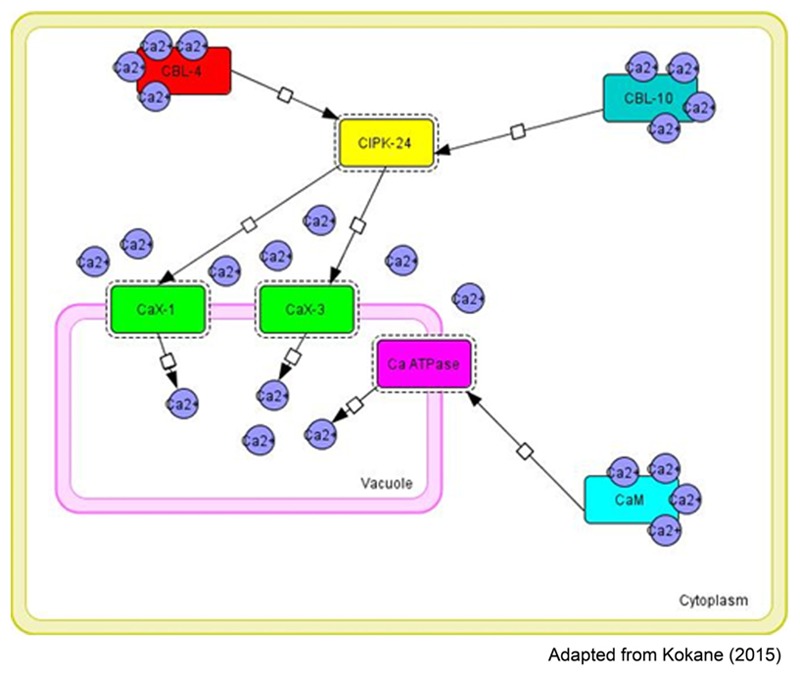
**Tripartite molecular interactions map using system biology graphical notation (SBGN) of the calcium exchangers and sensors in different tissues [CBL-4-CIPK24_CaX1 (Vegetative tissue), CBL-10-CIPK24-CaX1; CBL10-CIPK24-CaX3 (Vegetative tissue or developing spikes)] involved in the regulation of calcium transport and accumulation in finger millet (constructed by CellDesigner4.4)**.

Transcriptome sequence data of finger millet showed high expression of Ca^2+^ transporters including *CAX-1, CAX-3* and calcium sensor protein such as CIPK-24 and CaM in high seed calcium finger millet genotype (GPHCPB45). Transcript profiling (qPCR) study of such identified candidate genes indicated that Ca^2+^ transporters including *CAX-1, CAX-3* and calcium sensor proteins such as CIPK-8 and CaM are highly expressed in high seed calcium finger millet genotype (GPHCPB45) than GPHCPB1. Based on expression analysis and insilico interaction studies a speculative model is shown in **Figure 5** which shows tri-partite interaction of calcium transport and sensor genes leading to activation of transport machinery that pumps calcium from cytosol to vacuole in developing seeds in finger millet. It is suggested that SOS3/CBL4 and SOS2/CBL10 strongly interact with CIPK-24 in either vegetative or developing spikes stage. Such SOS/CBL complexes bind and activate CIPK-24 kinase activity by relieving self-inhibitory folding within the regulatory and kinase domain of the CIPK-24 protein (Halfter et al., 2000) and thus modulating the efficiency of CAX1 and CAX3 channel transporters. Ion transporters present at the tonoplast, including an H^+^ pump and Ca^2+^/H^+^ exchanger are the targets of CIPK-24 for their activation (Verslues et al., 2007). The calcium transport machinery regulated by calmodulin dependent and calmodulin independent pathways operative in seed tissues is leading to not only differential accumulation of calcium in seeds but also varied accumulation of calcium in different seed tissues viz. higher accumulation in aleurone layer, followed by seed coat and least in endosperm as reported by SEM-DEX analysis (Nath et al., 2012).

## Molecular Approaches for Calcium Biofortification

### Marker Discovery and Omics Approaches to Study the Molecular Basis of Calcium Accumulation

**FIGURE 6:**
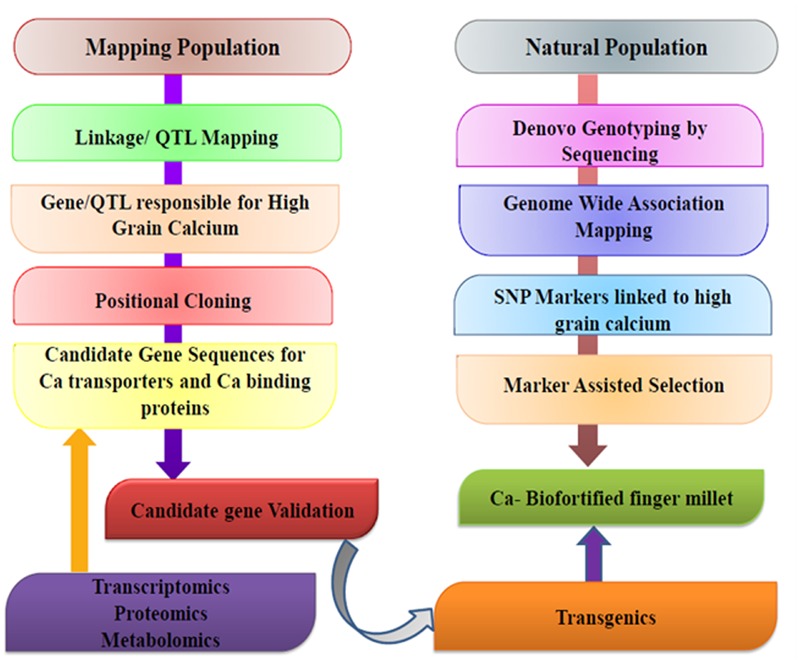
**Schematic representation of genomics and molecular breeding approaches for developing calcium biofortified finger millet**.

Fr From the last 20 years, molecular biology has revolutionized conventional breeding techniques in all areas. Biochemical and molecular techniques have shortened the duration of breeding programs from years to months or eliminated the need for them all together. The use of molecular markers in conventional breeding techniques have also improved the accuracy of crosses and allowed breeders to produce genotypes with combined traits that were very difficult before the advent of DNA technology. DNA markers can be generated in large numbers and can prove to be very useful for a variety of purposes relevant to crop improvement. **Figure 6** gives a schematic representation of genomics and molecular breeding approaches for developing calcium biofortified finger millet.

Low level of polymorphism has been reported in most of the studies on diversity analysis using molecular markers in cultivated finger millet (Muza et al., 1995). For the first time Dida et al. (2007) developed genomic SSRs by isolating di- and trinucleotide SSRs from random genomic *Hin*dIII, *Pst*I and *Sal*I libraries of finger millet. They developed first genetic map of finger millet with 31 genomic SSRs as well as RFLP, AFLP and EST markers. The sudden increase in the volume of sequence data generated from EST projects in several plant species facilitated the identification of genic SSRs in large numbers. Since the genomes of minor grasses like finger millet is yet to be sequenced, markers in minor grasses can be fetched from the major cereals. Comparative genetic mapping of cereal crops has shown that both gene contents and/or gene orders are largely conserved over the evolutionary history of the grasses (Moore et al., 1995) to the extent that grass genomes represent a ‘single genetic system’ (Bennetzen and Freeling, 1997).

Assessment of genetic diversity in finger millet revealed important information that South Indian and the African genotypes are close together and genetically distinct from North Indian genotypes including Uttarakhand (Panwar et al., 2010). Some of these genotypes of this crop possess very high grain calcium (Ca^2+^) content (450 mg/100 g) which is 10–30 times higher than wheat and rice (Panwar et al., 2010). In a study of Uttarakhand finger millet genotypes, molecular marker analysis differentiated the genotypes into three distinct clusters according to calcium (Ca^2+^) content indicating that variation in calcium content is also genetically controlled (Panwar et al., 2010). Markers have been utilized extensively for marker assisted selection, based on their association with genes/QTLs controlling grain calcium trait (**Table 2**). In order to identify the markers associated with high grain calcium trait, 146 genic SSR markers were assessed for cross species transferability across a diverse panel of grass species. The average transferability of genic SSR markers from sorghum to other grasses was highest (73.2%) followed by rice (63.4%) with an overall average of 68.3% which establishes the importance of these major crops as a useful resource of genomic information for minor crops. The genic SSR primers (69.7%) failed to detect variations across the finger millet germplasm, indicating that the mineral transport and storage machinery remain conserved in plants and even SSR variations in them remain suppressed during the course of evolution (Yadav et al., 2014). Development and molecular characterization of genic molecular markers for grain protein and calcium content has also been done (Nirgude et al., 2014). Of the 86 SSRs used in linkage mapping study, only 6 primers were polymorphic among the two parents PRM 801 (low calcium genotype) and GE 86 (high calcium genotype). Further, 20 polymorphic primers used across the association mapping panel of 238 genotypes led to the identification of 5 SSR markers viz. ugep67, ugep24, ugep77, ugep12 and ugep 10, which were significantly associated with calcium trait. For identifying QTLs for calcium content marker trait associations have been explored through association mapping studies and two minor QTLs associated with grain calcium (Ca^2+^) content on linkage group 3 and 8 respectively have been identified (Yadav et al., 2014). Linkage group 8 has been found to harbor a minor QTL for the trait and high levels of conserved co-linearity between rice and finger millet genomes (Srinivasachary et al., 2007) reveals that finger millet chromosome 8 might also contain genes/regions responsible for effective mineral accumulation. Similarly, finger millet LG 3 shares co-linearity with rice chromosome 3 and Ca^2+^ QTLs have also been mapped on chromosome 3 of rice. Furthermore, these results indicate that broad genome-wide search will be required to identify all the genes that control this complex trait and variation in a population.

**Table 2:** Molecular marker studies in Finger millet for calcium trait.

Source for designing primers	Primers	Polymorphism	Reference
Calcium(Ca^2+^) transporters and sensors of rice and sorghum	23 anchored EST SSRs	14 polymorphic markers	Kumar et al., 2015c
Calcium(Ca^2+^) transporters and sensors of rice and sorghum	146 EST SSRs	No polymorphism	Yadav et al., 2014
Candidate genes viz., Calcium(Ca^2+^) exchangers, channels and ATPases of finger millet, rice, maize, wheat and barley	20 anchored SSRs	5 polymorphic markers	Nirgude et al., 2014

The putative QTLs for calcium accumulation and increased calcium uptake have been explored in several plant species by different workers (Guzmán-Maldonado et al., 2003; Lexer et al., 2003; Zhang et al., 2009). In rice, QTLs for calcium accumulation have been identified and mapped on chromosomes 1, 3, 4, 5, 9, 10, 11, and 12 with phenotypic variation ranging from 5 to 18% in rice (Garcia-Oliveira et al., 2009; Du et al., 2013). Owing to sufficient variation for calcium content in finger millet germplasm, putative QTLs could be located using comparative genomics approach due to high co-linearity between rice and finger millet genomes.

One of the greatest challenges of biology in the post genomic era understands the functional connections between genes, transcripts, proteins, metabolites and nutrients (Kumar et al., 2015a). This understanding will highly beneficial for the plant biofortification processes. Storage of nutrients in different plant organs such as leaves, roots or seeds is governed by a specific subset of genes which ultimately control whole plant concentrations for numerous elements. The field of genomics has significantly contributed to this information, as it has helped in identifying the genes and genomes of several food plants. As it is already known that some genes are responsible for the uptake and transport of more than one mineral, thus, affecting the mineral homeostasis in a coordinated way. Therefore, alternate tools for expediting the prioritization of candidate genes that control mineral accumulation in plants are desirable (Conn et al., 2012). Therefore, the field of high throughput sequencing/transcriptomics has been fundamental in accelerating gene discovery.

Since, the finger millet crop is valued for its high calcium content, studies have been focused to characterize calcium (Ca^2+^) sensing, transport and accumulation mechanisms across genotypes differing in their grain calcium (Ca^2+^) content with the use of high-throughput transcriptome sequencing (Kumar et al., 2014; Singh et al., 2014). To generate the nucleotide sequence information resource and gain deep insights about the developing grain transcriptome and identify the genes responsible or involved in the process of high grain calcium (Ca^2+^) accumulation, Kumar et al. (2015b) used Illumina HiSeq-2000 high-throughput RNA sequencing technology to identify differentially expressed targets for calcium (Ca^2+^) content variation in two contrasting finger millet genotypes differing in grain calcium (Ca^2+^) content (GPHCPB45 – a high calcium genotype and GPHCPB1 – a low calcium genotype). The differential expression analysis on the basis of FPKM value resulted in 24 genes highly expressed in GPHCPB45 genotype and 11 were highly expressed in GPHCPB1 genes (Kumar et al., 2015b). The results obtained from transcriptome analysis were validated on spikes of finger millet grown on different concentration of calcium (Ca^2+^) (0.1, 5.0, 10, and 20 mM) at four stages of spike development. Significant correlation between the expression of selected Ca^2+^ sensor genes and amount of exogenous Ca^2+^ supply was observed (Singh et al., 2014).

Through peptide mass finger printing, two Ca^2+^ binding protein viz., ‘Calcineurin-B’ and ‘Calreticulin’ were identified in finger millet seeds (Singh et al., 2016). Following the information about the involvement of calcineurin and calreticulin proteins in binding calcium (Ca^2+^) in seed, expressions of 5 each calcineurin and calreticulin genes of finger millet were studied during grain filling. *EcCRT1*, which shows high expression in the S1 stage of seed development, was partially cloned, sequenced and its full length cloning is under progress (Singh et al., 2014). The mechanism of how Ca^2+^ are moving/transporting inside seed is still not known, as Ca^2+^ in phloem is immobile and seed is mainly fed by phloem. The preliminary results based on rice Ca^2+^ sensor and transporter data suggest that Ca^2+^ transporters (especially Ca Exchanger) are the main Ca^2+^ transporting proteins that pump Ca^2+^ inside seed (Li, 2006). The activities of these Ca^2+^ transporters are governed by Ca^2+^ sensor proteins such as CaM. Southern hybridization results showed the presence of at least four copies of the previously isolated *CaM*, that is located on different regions of the finger millet genome. Immuno-detection using mono-specific polyclonal anti-EcCaM antibodies revealed that EcCaM is localized in the embryo, close to the aleurone layer and accumulates in higher amounts in the high grain calcium genotype. Abundance of CaM around aleurone layer in high grain Ca^2+^ genotype suggests that CaM might be responsible for high grain calcium accumulation (Kumar et al., 2014). However, all studies on Ca^2+^ sensor and transporter genes were based on cloning of gene by designing primers from conserved region of related species/genus that may miss important gene of major effects. A detailed transcriptome wide study of calcium (Ca^2+^) sensors and transporters was carried out to identify the differentially expressed genes during developing spikes of finger millet and can serve as potential candidate genes having role in seed calcium accumulation (Singh et al., 2014, 2016). Transgenics for Ca^2+^ Exchanger (*CAX*), an important Ca^2+^ transporter that pump Ca^2+^ inside vacuoles were developed, which resulted in significant Ca^2+^ increase but deleterious effects like mineral imbalance, stunted plant growth and other structural changes were also seen (Kumar et al., 2015a). GPHCPB45 was found to be more efficient calcium (Ca^2+^) accumulator at low external calcium (Ca^2+^) concentrations, whereas GPHCPB1 was found to be more responsive to increased external calcium (Ca^2+^) concentration (Singh et al., 2014). Comprehensive sequencing efforts and analysis of gene function in the developing spikes transcriptome of finger millet (*E*. *coracana*) represents the most extensive expressed sequence resource available for finger millet to date (Kumar et al., 2014). Analysis of transcriptome sequence data of finger millet has indicated that Ca^2+^ transporter genes including *CAX-1, CAX-3* and calcium (Ca^2+^) sensor protein such as CIPK-24 and CaM are highly expressed in high seed Ca^2+^ finger millet genotype. In order to understand the role and interrelationship expression of these genes in high seed calcium (Ca^2+^) accumulation, comparative tissue wide transcript profiling study of potential candidate genes, viz. *CAX-1, CAX-3, CIPK-24* and *CaM* at vegetative stage and developing spikes stage have shown mostly higher expression in high grain calcium (Ca^2+^) genotype. The up-regulation of *CAX-1* in vegetative tissues and developing spikes and *CAX-3* in developing spikes provide most plausible clue explaining the accumulation of high calcium (Ca^2+^) in finger millet. The genetic information generated from these studies can be deployed in calcium (Ca^2+^) biofortification program by using genetic engineering or marker assisted selection strategies. Breeding for high grain Ca^2+^ in finger millet can be accelerated by using the closely linked markers to identified genes (*EcCIPKs*). This is just beginning, and it needs further studies in cloning and validation of the above genes using a set of different contrasting genotypes.

Recently, an effort has been also made to study the genetic make of diverse world finger millet collection and population structure analysis of 113 finger millet accessions using genotyping by sequencing (GBS) (Kumar et al., 2016). This resulted in a genome wide set of 23000 Single Nucleotide Polymorphisms (SNPs) segregating across the entire collection and several thousand SNPs segregating within every accession. It will provide a better opportunity to dissect complex traits and identification of superior alleles/ genes contributing to the target traits.

Considering the examination of mineral/micronutrients and their complex networks, ionomics or ionome profiling has emerged as a striking area that facilitates the genome-scale understanding of dynamics of elemental accumulation in the living systems (Baxter, 2010). In the context to ionomics, several techniques driven by the electronic or nuclear property of the element are currently being used to investigate elemental composition in different organisms (Singh et al., 2013). These techniques include AAS, ion beam analysis (IBA), X-ray fluorescence spectroscopy (XRF), ICP mass spectroscopy (ICP-MS) and neutron activation analysis (NAA) (Pfeiffer and McClafferty, 2007; Djingove et al., 2013; Singh et al., 2013).

Metabolomics is another area to better explore the chemical components which are present in food grains, how they are synthesized and the genetic and environmental factors that are involved in determining food composition and stability. Metabolomics is much more than just a diagnostics tool (Schauer and Fernie, 2006) and can help us greatly to improve our understanding of the complexity of metabolic regulation and explain how minor perturbations can have a myriad of biochemical end-points. This creates the potential to provide us with the knowledge necessary to facilitate a more targeted approach to crop improvement, specifically in terms of biochemical composition and nutritional value (Robert and Jennifer, 2010**).**

## Transgenic Technology

Transgenic may provide an alternative approach for biofortification of major food to enhance the concentration of calcium (Ca^2+^) content. The major aim of calcium biofortification strategy is to increase calcium (Ca^2+^) content in edible parts of crops without adversely affecting plant growth or increasing the plant requirement for additional inputs, i.e., calcium (Ca^2+^) fertilization or increased water use (Dayod et al., 2010). In plant three transgenic approaches can be used to increase the concentration of calcium (Ca^2+^) in edible part. The first approach is to enhance calcium (Ca^2+^) storage capacity through expression of calcium (Ca^2+^) transporter proteins viz., Ca^2+^ ATPase and Ca^2+^/H^+^ antiporters. Ca^2+^ ATPase requires energy for calcium (Ca^2+^) transport in the form of ATP while Ca^2+^/H^+^ antiporters is activated by proton motive force. Lower energy demands of transport through Ca^2+^/H^+^ antiporters make them a good candidate for Ca^2+^ increase (Conn and Gilliham, 2010). *Arabidopsis thaliana CAX1* (for CA*LCIUM E*X*CHANGER* 1) gene was first over expressed in tobacco plant under the control of cauliflower mosaic virus 35S promoter and 100% increase in calcium content was observed (Hirschi, 1999). Hirschi and colleagues expressed Yeast vacuolar Ca^2+^/H^+^ antiporter, (*VCX1*) in Arabidopsis and tobacco (Hirschi, 2001). VCX1-expressing plants demonstrated increased tonoplast-enriched Ca^2+^/H^+^ antiport activity as well as increased Ca^2+^ accumulation (upto 50%). Later several transgenic event were attempted by expression of different CAX proteins *viz*., *AtsCAX1* in carrot (Park et al., 2004; Morris et al., 2008), rice (Kim et al., 2006; Yi et al., 2012), potato (Park et al., 2005a), tomato (Park et al., 2005b), tobacco (Mei et al., 2007; Park et al., 2009) lettuce (Park et al., 2009); *AtCAX4* in tomato (Park et al., 2005b), *AtsCAX2B* in potato (Kim et al., 2006); *AtsCAX2a* in tomato (Chung et al., 2010); *AtsCAX2b* in bottle guard (Han et al., 2009) and upto 300% calcium (Ca^2+^) increase were reported. However, tissue Ca^2+^ increase was negatively co-related with Ca^2+^ deficiency symptoms. Interestingly, over expression of *AtCAX4* or modified *AtsCAX2B* have shown weaker Ca^2+^ transport and fewer deficiency symptoms (Park et al., 2005b; Chung et al., 2010). This indicates that *AtsCAX1* is too strong in pulling cytosolic Ca^2+^ away from cytoplasm and makes it unavailable for plant cellular processes than weaker alleles like *AtCAX4, AtsCAX2B*. Increasing bioavailable Ca^2+^, CAX transporters are excellent candidates but measure has to taken to avoid Ca^2+^ deficiency symptoms. Moreover, over expression of some CAX have shown little accumulation of other ions like Cd (cadmium), Mn (magnese) modification of the region could be used to increase specificity (Shigaki et al., 2003).

The second approach is to enhance calcium (Ca^2+^) accumulation by over expression of calcium (Ca^2+^) channel proteins. Calcium (Ca^2+^) channels in plasma membrane have been reported in all known cell types (Véry and Sentenac, 2002) but there are few reports on the genes that encode these proteins. GLR Proteins (Gilliham et al., 2006) and cyclic nucleotide gated channels (Demidchik and Maathuis, 2007) are a few among the candidate genes. Influx of calcium (Ca^2+^) across the PM is passive (i.e., down the Ca^2+^ electrochemical gradient) and is driven by Ca^2+^ channels (White and Broadley, 2003). The wheat low-affinity cation transporter gene (*LCT1*) was expressed in tobacco under the control of CaMV 35S promoter. LCT1-transformed plants expressed a phenotype distinct from controls only under conditions of low calcium, they grew significantly better and had slightly higher shoot calcium concentration (Antosiewicz and Hennig, 2004).

The third approach to enhance calcium (Ca^2+^) content in plant was by over expression of calcium (Ca^2+^) binding protein. The use of calcium (Ca^2+^) binding proteins might be very efficient way to increase calcium (Ca^2+^) accumulation in tissues. The over expression of *Zea mays* calreticulin (an ER-localized calcium binding protein) in Arabidopsis resulted in bioavailable calcium (Ca^2+^) increase in plant (Wyatt et al., 2002). Upto 9–35% increase of calcium (Ca^2+^) content was measured for induced transformants compared to controls. The use of calcium binding protein as candidate for Ca^2+^ biofortification is good but compared to CAX transporter very less increase of Ca^2+^ has been reported (Wyatt et al., 2002).

The fourth approach was through mutagenesis. Model forage crop *Medicago truncatula* was mutagenised with EMS and mutant with no (cod5) or reduced (cod6) calcium oxalate crystals were identified. In this report 10% reduction in calcium(Ca^2+^) content but 23% increase in calcium(Ca^2+^) absorbed by mice in feeding trial was observed (Morris et al., 2008). Out of four transgenic approach for calcium(Ca^2+^) biofortification in plants, maximum calcium(Ca^2+^) increase (up to 300% time) has been reported in potato over expressing modified *Arabidopsis AtsCAX1* protein but the bioavailability of calcium(Ca^2+^) was found with calreticulin gene (**Table 3**).

**Table 3:** List of events made toward calcium biofortification.

Source and name of transporter	Target plant	% Fold increase	Remarks	Reference
*Arabidopsis thaliana* (AtCAX1)	Tobacco (*Nicotiana tobacum*)	100% increase	Symptoms of Ca^2+^ deficiencies, ion imbalance	Hirschi, 1999
Yeast vacuolar Ca^2+^/H^+^ antiporter, (VCX1)	*Arabidopsis* and tobacco	50% increase	Sensitivity to Na^+^ and other ions	Hirschi, 2001
*Zea mays* calreticulin	*Arabidopsis thaliana*	9–35% increase	–	Wyatt et al., 2002
*Triticum aestivum* (LCT1)	Tobacco (*Nicotiana tobacum*)	–	Protective against Cd^2+^ toxicity	Antosiewicz and Hennig, 2004
*Arabidopsis thaliana* (AtsCAX1)	Carrot taproot (*Daucus carota*)	Twofold increase	Indistinguishable from wild type	Park et al., 2004; Morris et al., 2008
*Arabidopsis thaliana* (AtsCAX1)	Rice (*Oryza japonica*)	–	Indistinguishable from wild type	Kim et al., 2005
*Arabidopsis thaliana* (AtsCAX1)	Potato (*Solanum tuberosum*)	300% increase	Indistinguishable from wild type	Park et al., 2005a
*Arabidopsis thaliana* (AtsCAX1)	Tomato (*Lycopersicon esculentum*)	20–150% increase	Also increases in Cu^2+^, Fe^3+^, Mg^2+^, Mn^2+^ and Zn^2+^	Park et al., 2005b
*Arabidopsis thaliana* (AtCAX4)	Tomato (*Lycopersicon esculentum*)	40–50% increase	Indistinguishable from wild type	Park et al., 2005b
*Arabidopsis thaliana* (AtsCAX2B)	Potato (*Solanum tuberosum*)	50–65% increase	Indistinguishable from wild type	Kim et al., 2006
*Arabidopsis thaliana* (AtsCAX1)	Tobacco (*Nicotiana tobacum*)	200% increase	Also increase in K^+^ and Mn^2+^	Mei et al., 2007
*Arabidopsis thaliana* (AtsCAX1)	Tobacco (*Nicotiana tobacum*)	15% increase	–	Park et al., 2009
*Arabidopsis thaliana* (AtsCAX1)	Lettuce (*Lactuca sativa*)	25–32% increase	Indistinguishable from wild type	Park et al., 2009
*Arabidopsis thaliana* (AtsCAX2b)	Bottle gourd (*Lagenaria siceraria*)	9% increase	Also increase in Na^+^ and K^+^	Han et al., 2009
*Arabidopsis thaliana* (AtsCAX2a)	Tomato (*Lycopersicon esculentum*)	100% increase	Indistinguishable from wild type	Chung et al., 2010
*Arabidopsis thaliana* (AtCAX1)	Rice	Up to 2.4 time increase	Architectural variation in starch granule formation	Yi et al. (2012)

## Conclusion

Hi- throughput technologies of genomics, transcriptomics, proteomics, metabolomics and ionomics have shifted the focus from single gene research to a holistic understanding of gene function. None of the methods used in isolation provides enough information to infer function of an unknown gene, instead combined data from different functional genomics tools bring us close to this goal. Potential genes involved in high grain calcium (Ca^2+^) accumulation in finger millet once identified through these approaches can be validated by over expressing them through transgenics and subsequently the candidate genes may possibly be used to genetically modify crops and help them increase their grain calcium content. Genome wide variant detection in crops like finger millet is a preliminary step toward linking genotypic variation and phenotypes. The conversion of these genetic variants (the most prevalent of these being SNPs) into genetic markers is particularly important in agronomically valuable crops to allow for effective marker assisted selection strategies, map based gene cloning, whole genome fingerprinting, association studies and population based analyses. Toward these goals, in the absence of the genome sequence of finger millet an increasing number of large scale genetic variant discovery initiatives are being taken in conjunction with NGS platforms, allowing for drastically quicker and cheaper variant discovery, and leading toward a far more comprehensive view of the genome or transcriptome.

## Future Prospects

Genomics information has not only helped in efficient understanding of structural and functional aspects of many plant genomes but also has provided a feasible platform for manipulation of genomes for crop improvement. The near-future completion of genome sequencing project of finger millet will help in determining the function of every gene and ultimately how genes interact to form the basis of complex traits such as calcium nutrition. Finger millet is hence, no more called as a *coarse cereal* rather referred to as a nutri-cereal or nutraceutical. The properties on the whole make finger millet an ideal model for studying genomics and an implausible source for gene mining for complex traits such as grain calcium (Ca^2+^) content. Potential candidate genes responsible for high grain calcium (Ca^2+^) accumulation isolated from finger millet can help in improving other cereal crops through functional genomics and molecular breeding approaches and pave way for the development of designer crops.

## Author Contributions

AK conceptualized the manuscript. DS, GJ, and US wrote the manuscript. DS and SS assisted, edited and updated the manuscript. AK contributed critically in revising the draft and updating the manuscript for publication.

## Conflict of Interest Statement

The authors declare that the research was conducted in the absence of any commercial or financial relationships that could be construed as a potential conflict of interest.

The reviewer TB and handling Editor declared their shared affiliation, and the handling Editor states that the process nevertheless met the standards of a fair and objective review.

## References

[B1] Admassu S. Teamir M. Alemu D. (2009). Chemical composition of local and improved finger millet (*Eleusine coracana* (L.) Gaetrtin) varieties grown in Ethiopia. *Ethiop. J. Health Sci.* 19 1–8.

[B2] Amtmann A. Blatt M. R. (2009). Regulation of macronutrient transport. *New Phytol.* 81 35–52. 10.1111/j.1469-8137.2008.02666.x 19076716

[B3] Anonymous (1996). *European Pharmacopoeia* 3rd Edn. Strasburg: Council of Europe 121–122.

[B4] Antony U. Chandra T. S. (1999). Enzymatic treatment and use of starters for the nutrient enhancement in fermented flour of red and white varieties of finger millet (*Eleusine coracana*). *J. Agric. Food Chem.* 47 2016–2019. 10.1021/jf980564a 10552488

[B5] Antosiewicz D. M. Hennig J. (2004). Overexpression of LCT1 in tobacco enhances the protective action of calcium against cadmium toxicity. *Environ. Pollut.* 129 237–245. 10.1016/j.envpol.2003.10.025 14987809

[B6] Atkinson C. J. Ruiz L. P. Mansfield T. A. (1992). Calcium in xylem sap and the regulation of its delivery to the shoot. *J. Exp. Bot.* 43 1315–1324. 10.1093/jxb/43.10.1315

[B7] Babu B. V. Ramana T. Radhakrishnan T. M. (1987). Chemical composition and protein content in hybrid varieties of finger millet. *Indian J. Agric. Sci.* 57 520–522.

[B8] Barbeau W. E. Hilu K. W. (1993). Protein, calcium, iron, and amino acid content of selected wild and domesticated cultivars of finger millet. *Plant Foods Hum. Nutr.* 43 97–104. 10.1007/BF01087914 8475005

[B9] Barnabas A. B. Arnott H. J. (1990). Calcium oxalate crystal formation in the bean (*Phaseolus vulgaris* L.) seed coat. *Bot. Gaz.* 151 331–341. 10.1086/337833

[B10] Baxter I. (2010). Ionomics: the functional genomics of elements. *Brief. Funct. Genomics* 9 149–156. 10.1093/bfgp/elp055 20081216

[B11] Baxter I. Tchieu J. Sussman M. R. Boutry M. Palmgren M. G. Gribskov M. (2003). Genomic comparison of P-type ATPase ion pumps in *Arabidopsis* and rice. *Plant Physiol*. 132 618–628. 10.1104/pp.103.021923 12805592 PMC167002

[B12] Bennett B. Sammartano R. (2012). *The Complete Idiot’s Guide to Vegan Living* Second Edn. Sittingbourne: Health and Fitness.

[B13] Bennetzen J. L. Freeling M. (1997). The unified grass genome: synergy in synteny. *Genome Res.* 7 301–306. 9110169 10.1101/gr.7.4.301

[B14] Bhatia V. (2008). Dietary calcium intake-a critical reappraisal. *Indian J. Med. Res.* 127:269. 18497442

[B15] Bhatt A. Singh V. Shrotria P. K. Baskheti D. C. (2003). Coarse grains of uttaranchal: ensuring sustainable food and nutritional security. *Indian Farmer Digest* 7 34–38.

[B16] Blumwald E. Poole R. J. (1986). Kinetics of Ca2+/H+ antiport in isolated tonoplast vesicles from storage tissue of *Beta vulgaris*. *Plant Physiol.* 80 727–731. 10.1104/pp.80.3.727 16664693 PMC1075191

[B17] Bouis H. E. (2003). Micronutrient fortification of plants through plant breeding: can it improve nutrition in man at low cost? *Proc. Nutr. Soc.* 62 403–411. 10.1079/PNS2003262 14506888

[B18] Canny M. J. (1993). The transpiration stream in the leaf apoplast-water and solutes. *Philos. Trans. R. Soc. B* 341 87–100. 10.1098/rstb.1993.0094

[B19] Carter C. Pan S. Zouhar J. Avila E. L. Girke T. Raikhel N. V. (2004). The vegetative vacuole proteome of *Arabidopsis thaliana* reveals predicted and unexpected proteins. *Plant Cell* 16 3285–3303. 10.1105/tpc.104.027078 15539469 PMC535874

[B20] Carvalho S. M. Vasconcelos M. W. (2013). Producing more with less: strategies and novel technologies for plant-based food biofortification. *Food Res. Int.* 54 961–971. 10.1016/j.foodres.2012.12.021

[B21] Chan B. K. Marshall L. M. Winters K. M. Faulkner K. A. Schwartz A. V. Orwoll E. S. (2007). Incident fall risk and physical activity and physical performance among older men the osteoporotic fractures in men study. *Am. J. Epidemiol.* 165 696–703. 10.1093/aje/kwk050 17194749

[B22] Chan G. M. McElligott K. McNaught T. Gill G. (2006). Effects of dietary calcium intervention on adolescent mothers and newborns: a randomized controlled trial. *Obstet. Gynecol.* 108 565–571. 10.1097/01.AOG.0000231721.42823.9e 16946216

[B23] Chethan S. Malleshi N. G. (2007). Finger millet polyphenols: characterization and their nutraceutical potential. *Am. J. Food Technol.* 2 582–592. 10.3923/ajft.2007.582.592

[B24] Chung M. Y. Han J. S. Giovannoni J. Liu Y. Kim C. K. Lim K. B. (2010). Modest calcium increase in tomatoes expressing a variant of *Arabidopsis* cation/H+ antiporter. *Plant Biotech Rep.* 4 15–21. 10.1007/s11816-009-0112-9

[B25] Cocozza C. Minnocci A. Tognetti R. Iori V. Zacchini M. Scarascia Mugnozza G. (2008). Distribution and concentration of cadmium in root tissue of Populus alba determined by scanning electron microscopy and energy-dispersive x-ray microanalysis. *iFor. Biogeosci. For.* 1 96–103. 10.3832/ifor0458-0010096

[B26] Collard B. C. Mackill D. J. (2008). Marker-assisted selection: an approach for precision plant breeding in the twenty-first century. *Philos. Trans. R. Soc. B Biol. Sci.* 12 363 557–572. 10.1098/rstb.2007.2170 PMC2610170 17715053

[B27] Conn S. Gilliham M. (2010). Comparative physiology of elemental distributions in plants. *Ann. Bot.* 105 1081–1102. 10.1093/aob/mcq027 20410048 PMC2887064

[B28] Conn S. J. Berninger P. Broadley M. R. Gilliham M. (2012). Exploiting natural variation to uncover candidate genes that control element accumulation in *Arabidopsis thaliana*. *New Phytol.* 193 859–866. 10.1111/j.1469-8137.2011.03977.x 22403822

[B29] Conn S. J. Gilliham M. Athman A. (2011). Cell-specific vacuolar calcium storage mediated by CAX1 regulates apoplastic calcium concentration, gas exchange, and plant productivity in *Arabidopsis*. *Plant Cell* 23 240–257. 10.1105/tpc.109.072769 21258004 PMC3051233

[B30] Crossa J. De los Campos G. Perez P. Gianola D. Burgueno J. Araus J. L. (2010). Prediction of genetic values of quantitative traits in plant breeding using pedigree and molecular markers. *Genetics* 186 713–724. 10.1534/genetics.110.118521 20813882 PMC2954475

[B31] Dayod M. Tyerman S. D. Leigh R. A. Gilliham M. (2010). Calcium storage in plants and the implications for calcium biofortification. *Protoplasma* 247 215–231. 10.1007/s00709-010-0182-0 20658253

[B32] Demidchik V. Maathuis F. J. (2007). Physiological roles of non selective cation channels in plants: from salt stress to signalling and development. *New Phytol.* 175 387–404. 10.1111/j.1469-8137.2007.02128.x 17635215

[B33] Deosthale Y. G. (2002). *The Nutritive Value of Foods and the Significance of some Household Processes*. Available at: http://www.unu.edu. p. 6

[B34] Devi P. B. Vijayabharathi R. Sathyabama S. Malleshi N. G. Priyadarisini V. B. (2014). Health benefits of finger millet (*Eleusine coracana* L.) polyphenols and dietary fiber: a review. *J. Food Sci. Technol.* 51 1021–1040. 10.1007/s13197-011-0584-9 24876635 PMC4033754

[B35] Dida M. M. Ramakrishnan S. Bennetzen J. L. Gale M. D. Devos K. M. (2007). The genetic map of finger millet, *Eleusine coracana*. *Theor. Appl. Genet.* 114 321–332. 10.1007/s00122-006-0435-7 17103137

[B36] Djingove R. Mihaylova V. Lyubomirova V. Tsalev D. L. (2013). Multi element analytical spectroscopy in plant ionomics research. *Appl. Spectrosc. Rev.* 48 384–424. 10.1080/05704928.2012.703153

[B37] Du J. Zeng D. Wang B. Qian Q. Zheng S. Ling H. Q. (2013). Environmental effects on mineral accumulation in rice grains and identification of ecological specific QTLs. *Environ. Geochem. Health* 35 161–170. 10.1007/s10653-012-9473-z 22760687

[B38] Fairweather-Tait S. Hurrell R. F. (1996). Bioavailability of minerals and trace elements. *Nutr. Res. Rev.* 9 295–324. 10.1079/NRR19960016 19094275

[B39] Food and Agriculture Organization [FAO] of the United Nations (2008). *Food* *Insecurity and Vulnerability Information and Mapping Systems (FIVMS)* Rome: FAO.

[B40] Food and Agriculture Organization [FAO] of the United Nations (2009). *2050: A Third more Mouths to Feed*. Rome: FAO.

[B41] Franceschi V. R. Nakata P. A. (2005). Calcium oxalate in plants: formation and function. *Annu. Rev. Plant Biol.* 56 41–71. 10.1146/annurev.arplant.56.032604.144106 15862089

[B42] Furuichi T. Cunningham K. W. Muto S. (2001). A putative two pore channel AtTPC1 mediates Ca^2+^ flux in *Arabidopsis* leaf cells. *Plant Cell Physiol*. 42 900–905. 10.1093/pcp/pce145 11577183

[B43] Garcia-Oliveira A. L. Tan L. Fu Y. Sun C. (2009). Genetic identification of quantitative trait loci for contents of mineral nutrients in rice grain. *J. Integr. Plant Biol.* 51 84–92. 10.1111/j.1744-7909.2008.00730.x 19166498

[B44] Genc Y. Humphries J. M. Lyons G. H. Graham R. D. (2005). Exploiting genotypic variation in plant nutrient accumulation to alleviate micronutrient deficiency in populations. *J. Trace Elem. Med. Biol.* 18 319–324. 10.1016/j.jtemb.2005.02.005 16028493

[B45] Gilliham M. Campbell M. Dubos C. Becker D. Davenport R. (2006). “The *Arabidopsis thaliana* glutamate-like receptor family (AtGLR),” in *Communication in Plants* eds Baluška F. Mancuso S. Volkmann D. (Berlin: Springer) 187–204.

[B46] Gilliham M. Dayod M. Hocking B. J. Xu B. Conn S. J. Kaiser B. N. (2011). Calcium delivery and storage in plant leaves: exploring the link with waterflow. *J. Exp. Bot.* 62 2233–2250. 10.1093/jxb/err111 21511913

[B47] Goel A. Gaur V. S. Arora S. Gupta S. Kumar A. (2012). In silico analysis of expression data for identification of genes involved in spatial accumulation of calcium in developing seeds of rice. *Omics* 16 402–413. 10.1089/omi.2012.0004 22734689 PMC3394857

[B48] Goel A. Taj G. Pandey D. Gupta S. Kumar A. (2011). Genome-wide comparative in silico analysis of calcium transporters of rice and sorghum. *Genomics Proteomics Bioinformatics* 9 138–150. 10.1016/S1672-0229(11)60017-X 22196357 PMC5054455

[B49] Gopalan C. Ramshashtri B. V. Balasubramanian S. C. (1999). *Nutritive Value of Indian Foods* eds NarasingaRao B. S. Deosthale Y. G. Pant K. C. Hyderabad: National Institute of Nutrition 156.

[B50] Govindaraj M. Vetriventhan M. Srinivasan M. (2015). Importance of genetic diversity assessment in crop plants and its recent advances: an overview of its analytical perspectives. *Genet. Res. Int.* 2015:431487 10.1155/2015/431487 PMC4383386 25874132

[B51] Graham R. D. Welch R. M. Bouis H. E. (2001). Addressing micronutrient malnutrition through enhancing the nutritional quality of staple foods: principles, perspectives and knowledge gaps. *Adv. Agron.* 70 77–142. 10.1016/S0065-2113(01)70004-1

[B52] Guo Z. A. Song Y. X. Zhou R. H. Ren Z. L. Jia J. Z. (2010). Discovery, evaluation and distribution of haplotypes of the wheat Ppd-D1 gene. *New Phytol.* 185 841–851. 10.1111/j.1469-8137.2009.03099.x 20002313

[B53] Guzmán-Maldonado S. H. Martinez O. Acosta-Gallegos J. A. Guevara-Lara F. Paredes-Lopez O. (2003). Putative quantitative trait loci for physical and chemical components of common bean. *Crop Sci.* 43 1029–1035. 10.2135/cropsci2003.1029

[B54] Halfter U. M. Ishitani M. Zhu J. K. (2000). The *arabidopsis* SOS2protein kinase physically interacts with and activated by the calcium binding protein SOS3. *Proc. Natl. Acad. Sci. U.S.A.* 97 3735–3740. 10.1073/pnas.97.7.3735 10725350 PMC16309

[B55] Han J. C. Yang X. D. Zhang T. Li H. Li W. L. Zhang Z. Y. (2009). Effects of 1alpha-hydroxycholecalciferol on growth performance, parameters of tibia and plasma, meat quality, and type IIb sodium phosphate co transporter gene expression of one- to twenty-one-day-old broilers. *Poult. Sci.* 88 323–329. 10.3382/ps.2008-00252 19151347

[B56] Haswell E. S. Peyronnet R. Barbier-Brygoo H. Meyerowitz E. M. Frachisse J. M. (2008). Two MscS homologs provide mechanosensitive channel activities in the *Arabidopsis* root. *Curr. Biol*. 18 730–734. 10.1016/j.cub.2008.04.039 18485707

[B57] Haug W. Lantzsch H. J. (1983). Sensitive method for the rapid determination of the phytate in cereals and cereal products. *J. Sci. Food Agric.* 34 1423–1426. 10.1002/jsfa.2740341217

[B58] Heaney R. P. (1993). Nutritional factors in osteoporosis. *Annu. Rev. Nutr.* 13 287–316. 10.1146/annurev.nu.13.070193.001443 8369148

[B59] Heffner E. L. Jannink J. L. Sorrells M. E. (2011). Genomic selection accuracy using multifamily prediction models in a wheat breeding program. *Plant Genome* 4 65–75. 10.3835/plantgenome.2010.12.0029

[B60] Hirschi K. D. (1999). Expression of *Arabidopsis* CAX1 in tobacco: altered calcium homeostasis and increased stress sensitivity. *Plant Cell* 11 2113–2122. 10.2307/3871013 10559438 PMC144126

[B61] Hirschi K. D. (2001). Vacuolar H+/Ca2+ transport: who’s directing the traffic? *Trends Plant Sci.* 6 100–104. 10.1016/S1360-1385(00)01863-X 11239607

[B62] Hirschi K. D. (2009). Nutrient biofortification of food crops. *Annu. Rev. Nutr.* 21 401–421. 10.1146/annurev-nutr-080508-141143 19400753

[B63] House W. A. (1999). Trace element bioavailability as exemplified by iron and zinc. *Field Crops Res.* 60 115–141. 10.1016/S0378-4290(98)00136-1

[B64] Ilarslan H. Palmer R. G. Horner H. T. (2001). Calcium oxalate crystals in developing seeds of soybean. *Ann. Bot.* 88 243–257. 10.1006/anbo.2001.1453 21708659

[B65] Islam M. M. Munemasa S. Hossain M. A. Nakamura Y. Mori I. C. Murata Y. (2010). Roles of AtTPC1 vacuolar two pore channel 1 in *Arabidopsis* stomatal closure. *Plant Cell Physiol*. 51 302–311. 10.1093/pcp/pcq001 20061305

[B66] Jeong J. Guerinot M. L. (2008). Biofortified and bioavailable: the gold standard for plant-based diets. *Proc. Natl. Acad. Sci. U.S.A.* 105 1777–1778. 10.1073/pnas.0712330105 18256182 PMC2538837

[B67] Kadota Y. Furuichi T. Ogasawara Y. Goh T. Higashi K. Muto S. (2004). Identification of putative voltage-dependent Ca^2+^-permeable channels involved in cryptogein-induced Ca^2+^ transients and defense responses in tobacco BY-2 cells. *Biochem. Biophys. Res. Commun*. 317 823–830. 10.1016/j.bbrc.2004.03.114 15081414

[B68] Kaplan B. Sherman T. Fromm H. (2007). Cyclic nucleotide-gated channels in plants. *FEBS Lett*. 581 2237–2246. 10.1016/j.febslet.2007.02.017 17321525

[B69] Karley A. J. White P. J. (2009). Moving cationic minerals to edible tissues: potassium, magnesium, calcium. *Curr. Opin. Plant Biol.* 12 291–298. 10.1016/j.pbi.2009.04.013 19481494

[B70] Kim C. K. Han J. S. Lee H. S. Oh J. Y. Shigaki T. Park S. H. (2006). Expression of an *Arabidopsis* CAX2 variant in potato tubers increases calcium levels with no accumulation of manganese. *Plant Cell Rep.* 25 1226–1232. 10.1007/s00299-006-0214-6 17024452

[B71] Kim K. M. Park Y. H. Kim C. K. Hirschi K. Sohn J. K. (2005). Development of transgenic rice plants overexpressing the *Arabidopsis* H^+^/Ca^2+^ antiporter CAX1 gene. *Plant Cell Rep*. 23 678–682. 10.1007/s00299-004-0861-4 15372195

[B72] Kokane S. (2015). *Tissue Wide Expression Analysis of Potential Candidate Genes Involved in Calcium Signaling and Transport in Finger Millet (Eleusine coracana) Genotypes Differing in Grain Calcium Content.* Master’s thesis, G. B Pant University of Agriculture and Technology Pantnagar.

[B73] Kranz S. Lin P. J. Wagstaff D. A. (2007). Children’s dairy intake in the United States: too little, too fat? *J. Paediatr.* 151 642–646. 10.1016/j.jpeds.2007.04.067 18035145

[B74] Kumar A. Gaur V. S. Goel A. Gupta A. K. (2015b). De novo assembly and characterization of developing spikes transcriptome of finger millet (*Eleusine coracana*): a minor crop having nutraceutical properties. *Plant Mol. Biol. Rep.* 33 905–922. 10.1007/s11105-014-0802-5

[B75] Kumar A. Mirza N. Charan T. Sharma N. Gaur V. S. (2014). Isolation, characterization and immunolocalization of a seed dominant CaM from finger millet (*Eleusinecoracana* L. Gartn.) for studying its functional role in differential accumulation of calcium in developing grains. *Appl. Biochem. Biotechnol.* 172 2955–2973. 10.1007/s12010-013-0714-0 24469585

[B76] Kumar A. Pathak R. K. Gupta S. M. Gaur V. S. Pandey D. (2015a). Systems biology for smart crops and agricultural innovation: filling the gaps between genotype and phenotype for complex traits linked with robust agricultural productivity and sustainability. *OMICS* 19 581–601. 10.1089/omi.2015.0106 26484978 PMC4617413

[B77] Kumar A. Sharma D. Tiwari A. Jaiswal J. P. Singh N. K. Sood S. (2016). Genotyping-by-sequencing analysis for determining population structure of finger millet germplasm of diverse origins. *Plant Genome* 9 10.3835/plantgenome2015.07.0058 27898819

[B78] Kumar A. Yadav S. Panwar P. Gaur V. S. Sood S. (2015c). Identification of anchored simple sequence repeat markers associated with calcium content in finger millet (*Eleusine coracana*). *Proc. Natl. Acad. Sci. India B Biol. Sci*. 85 311–317. 10.1007/s40011-013-0296-1

[B79] Kurusu T. Sakurai Y. Miyao A. Hirochika H. Kuchitsu K. (2004). Identification of a putative voltage-gated Ca^2+^-permeable channel (OsTPC1) involved in Ca^2+^ influx and regulation of growth and development in rice. *Plant Cell Physiol*. 45 693–702. 10.1093/pcp/pch082 15215504

[B80] Lacombe B. Becker D. Hedrich R. DeSalle R. Hollmann M. Kwak J. M. (2001). The identity of plant glutamate receptors. *Science* 292 1486–1487. 10.1126/science.292.5521.1486b 11379626

[B81] Lanham-New S. A. (2008). Importance of calcium, vitamin D and vitamin K for osteoporosis prevention and treatment. *Proc. Nutr. Soc.* 67 163–176. 10.1017/S0029665108007003 18412990

[B82] Lexer C. Welch M. E. Durphy J. L. Rieseberg L. H. (2003). Natural selection for salt tolerance quantitative trait loci (QTLs) in wild sunflower hybrids: implications for the origin of Helianthus paradoxus, a diploid hybrid species. *Mol. Ecol.* 12 1225–1235. 10.1046/j.1365-294X.2003.01803.x 12694286

[B83] Li X. (2006). *The Importance of Sorting Calcium in Plant Cells: Uncovering the Roles of A Sarcoplasmic/Endoplasmic Reticulum-Like Calcium ATPase*. Ph.D. thesis, University of Maryland College Park Maryland, MD.

[B84] Malleshi N. G. Klopfenstein C. F. (1998). Nutrient composition, amino acid and vitamin contents of malted sorghum, pearl millet, finger millet and their rootlets. *Int. J. Food Sci. Nutr.* 49 415–422. 10.3109/09637489809086420

[B85] Matuschek E. Towo E. Svanberg U. (2001). Oxidation of polyphenols in phytate-reduced high-tannin cereals: effect on different phenolic groups and on in vitro accessible iron. *J. Agric. Food Chem.* 49 5630–5638. 10.1021/jf0108157 11714370

[B86] McLaughlin S. B. Wimmer R. (1999). Calcium physiology and terrestrial ecosystem processes. *New Phytol.* 142 373–417. 10.1046/j.1469-8137.1999.00420.x

[B87] Mei Y. Yang H. Sun J. Yin X. Guo X. Wang Z. (2007). Effects of different dust particles on calcium current in the neurons of dorsal root ganglia of rats. *J. Life Sci.* 4 10–14.

[B88] Mirza N. Taj G. Arora S. Kumar A. (2014). Transcriptional expression analysis of genes involved in regulation of calcium translocation and storage in finger millet (*Eleusine coracana* L.G*artn.)*. *Gene* 550 171–179. 10.1016/j.gene.2014.08.005 25101868

[B89] Moore G. Devos K. M. Wang Z. Gale M. D. (1995). Cereal genome evolution: grasses, line up and form a circle. *Curr. Biol.* 5 737–739. 10.1016/S0960-9822(95)00148-5 7583118

[B90] Morris J. Hawthorne K. M. Hotze T. Abrams S. A. Hirschi K. D. (2008). Nutritional impact of elevated calcium transport activity in carrots. *Proc. Natl. Acad. Sci. U.S.A.* 105 1431–1435. 10.1073/pnas.0709005105 18202180 PMC2234161

[B91] Müller O. Krawinkel M. (2005). Malnutrition and health in developing countries. *Can. Med. Assoc. J.* 173 279–286. 10.1503/cmaj.050342 16076825 PMC1180662

[B92] Muza F. R. Lee D. J. Andrews D. J. Gupta S. C. (1995). Mitochondrial DNA variation in finger millet (*Eleusinecoracana* L. Gaertn). *Euphytica* 81 199–205. 10.1007/BF00025434

[B93] Narina S. S. Buyyarapu R. Kottapalli K. R. Sartie A. M. Ali M. I. Robert A. (2011). Generation and analysis of expressed sequence tags (ESTs) for marker development in yam (*Dioscoreaa lata* L.). *BMC Genomics.* 12:1 10.1186/1471-2164-12-100 PMC3047301 21303556

[B94] Nath M. Partha R. Shukla A. Kumar A. (2012). Spatial distribution and accumulation of calcium in different tissues, developing spikes and seeds of finger millet (*Eleusine coracana*) genotype. *J. Plant Nutr.* 36 539–550. 10.1080/01904167.2012.748072

[B95] Nestel P. Bouis H. E. Meenakshi J. V. Pfeiffer W. (2006). Biofortification of staple food crops. *J. Nutr.* 1 1064–1067. 10.1093/jn/136.4.1064 16549478

[B96] Nirgude M. Babu B. K. Shambhavi Y. Singh U. M. Upadhyaya H. D. Kumar A. (2014). Development and molecular characterization of genic molecular markers for grain protein and calcium content in finger millet (*Eleusine coracana* (L.) Gaertn.). *Mol. Biol. Rep.* 41 1189–1200. 10.1007/s11033-013-2825-7 24477581

[B97] Obilana A. B. Manyasa E. (2002). “Millets,” in *Pseudocereals and Less Common Cereals. Grain Properties and Utilization Potential* eds Belton P. S. Taylor J. R. N. (Berlin: Springer-Verlag) 176–217.

[B98] Panwar P. Nath M. Kumar V. Kumar A. (2010). Comparative evaluation of genetic diversity using RAPD, SSR and cytochrome P450 gene based markers with respect to calcium content in finger millet (*Eleusine coracana* L. Gaertn.). *J. Genet.* 89 121–133. 10.1007/s12041-010-0052-8 20861563

[B99] Park S. Cheng N. H. Pittman J. K. Yoo K. S. Park J. Smith R. H. (2005a). Increased calcium levels and prolonged shelf life in tomatoes expressing *Arabidopsis* H+/Ca2+ transporters. *Plant Physiol.* 139 1194–1206. 10.1104/pp.105.066266 16244156 PMC1283758

[B100] Park S. Elless M. P. Park J. Jenkins A. Lim W. Chambers E. (2009). Sensory analysis of calcium-biofortified lettuce. *Plant Biotechnol. J.* 7 106–111. 10.1111/j.1467-7652.2008.00379.x 19021875

[B101] Park S. Kang T. S. Kim C. K. Han J. S. Kim S. Smith R. H. (2005b). Genetic manipulation for enhancing calcium content in potato tuber. *J. Agric. Food Chem.* 53 5598–5603. 10.1021/jf050531c 15998121

[B102] Park S. Kim C. K. Pike L. M. Smith R. H. Hirschi K. D. (2004). Increased calcium in carrots by expression of an *Arabidopsis* H+/Ca2+ transporter. *Mol. Breed.* 14 275–282. 10.1023/B:MOLB.0000047773.20175.ae

[B103] Patrick J. W. (1990). Sieve element unloading: cellular pathway, mechanism and control. *Physiol. Plant.* 78 298–308. 10.1034/j.1399-3054.1990.780220.x

[B104] Patrick J. W. (1997). Phloem unloading: sieve element unloading and post-sieve element transport. *Annu. Rev. Plant Physiol. Plant Mol. Biol.* 48 191–222. 10.1146/annurev.arplant.48.1.191 15012262

[B105] Pettifor J. M. (2008). Vitamin D &/or calcium deficiency rickets in infants & children: a global perspective. *Indian J. Med. Res.* 127 245. 18497438

[B106] Pfeiffer W. H. McClafferty B. (2007). Harvest plus: breeding crops for better nutrition. *Crop Sci.* 47 88–105. 10.2135/cropsci2007.09.0020IPBS

[B107] Piepho H. P. (2009). Ridge regression and extensions for genomewide selection in maize. *Crop Sci.* 49 1165–1176. 10.2135/cropsci2008.10.0595

[B108] Pittman J. K. (2011). Vacuolar Ca^2+^ uptake. *Cell Calcium* 50 139–146. 10.1016/j.ceca.2011.01.004 21310481

[B109] Pravina P. Sayaji D. Avinash M. (2013). Calcium and its role in human body. *Int. J. Res. Pharm. Biomed. Sci.* 4 659–668.

[B110] Punshon T. Hirschi K. Yang J. Lanzirotti A. Lai B. Guerinot M. L. (2012). The role of CAX1 and CAX3 in elemental distribution and abundance in *Arabidopsis* seed. *Plant Physiol.* 158 352–362. 10.1104/pp.111.184812 22086421 PMC3252103

[B111] Raboy V. (2000). Low phytic acid grains. *Food Nutr. Bull.* 21 423–427. 10.1177/156482650002100416

[B112] Rao A. S. Prabhu U. H. Sampath S. R. Schiere J. B. (1994). The effect of level of allowance on the intake and digestibility of finger millet (*Eleusine coracana*) straw in crossbred heifers. *Anim. Feed Sci. Technol.* 49 37–41. 10.1016/0377-8401(94)90079-5

[B113] Rao M. V. S. S. T. S. Muralikrishna G. (2002). Evaluation of the antioxidant properties of free and bound phenolic acids from native and malted finger millet (ragi, *Eleusine coracana* Indaf-15). *J. Agric. Food Chem.* 50 889–892. 10.1021/jf011210d 11829663

[B114] Ravindran G. (1991). Studies on millets: proximate composition, mineral composition, phytate, and oxalate contents. *Food Chem.* 39 99–107. 10.1016/0308-8146(91)90088-6

[B115] Rengel Z. Graham R. D. (1995). Importance of seed zinc content for wheat growth on zinc-deficient soil. I. Vegetative growth. *Plant Soil* 173 259–266. 10.1007/BF00011463

[B116] Robert M. Y. Jennifer K. (2010). Systems mapping of consumer acceptance of agri food nanotechnology. *J. Consum. Policy.* 33 299–322. 10.1007/s10603-010-9134-5

[B117] Saied H. T. Shamsuddin A. M. (1998). Up-regulation of the tumour suppressor gene p53 and WAF1 gene expression by IP6 in HT-29 human colon carcinoma cell line. *Anticancer Res.* 18 1479–1484. 9673359

[B118] Schauer N. Fernie A. R. (2006). Plant metabolomics: towards biological function and mechanism. *Trends Plant Sci.* 11 508–516. 10.1016/j.tplants.2006.08.007 16949327

[B119] Seetharam A. (2001). *Annual Report: All India Coordinated Small Millets Improvement Project*. Bangalore: University of Agricultural Sciences 1–28.

[B120] Shamsuddin A. M. (1999). Metabolism and cellular functions of IP6: a review. *Anticancer Res.* 19 3733–3736. 10625949

[B121] Shigaki T. Pittman J. K. Hirschi K. D. (2003). Manganese specificity determinants in the *Arabidopsis* metal/H+ antiporter CAX2. *J. Biol. Chem.* 278 6610–6617. 10.1074/jbc.M209952200 12496310

[B122] Singh M. Metwal M. Kumar V. A. Kumar A. (2016). Identification and molecular characterization of 48 kDa calcium binding protein as calreticulin from finger millet (*Eleusine coracana*) using peptide mass fingerprinting and transcript profiling. *J. Sci. Food Agric.* 96 672–679. 10.1002/jsfa.7139 25684084

[B123] Singh P. Raghuvanshi R. S. (2012). Finger millet for food and nutritional security. *Afr. J. Food Sci.* 6 77–84.

[B124] Singh U. M. Metwal M. Singh M. Taj G. Kumar A. (2015). Identification and characterization of calcium transporter gene family in finger millet in relation to grain calcium content. *Gene* 566 37–46. 10.1016/j.gene.2015.04.021 25869323

[B125] Singh U. M. Pandey D. Kumar A. (2014). Determination of calcium responsiveness towards exogenous application in two genotypes of *Eleusine coracana* L. differing in their grain calcium content. *Acta Physiol. Plantarum.* 36 2521–2529. 10.1007/s11738-014-1625-6

[B126] Singh U. M. Sareen P. Sengar R. S. Kumar A. (2013). Plant ionomics: a newer approach to study mineral transport and its regulation. *Acta Physiol. Plant.* 35 2641–2653. 10.1007/s11738-013-1316-8

[B127] Srinivasachary Dida M. M. Gale M. D. Devos K. M. (2007). Comparative analyses reveal high levels of conserved colinearity between the finger millet and rice genomes. *Theor. Appl. Genet.* 115 489–499. 10.1007/s00122-007-0582-5 17619853

[B128] Timmer C. P. (2003). Biotechnology and food systems in developing countries. *J. Nutr.* 133 3319–3322. 14608038 10.1093/jn/133.11.3319

[B129] Underwood R. A. (2000). Overcoming micronutrient deficiencies in developing countries: is there a role for agriculture? *Food Nutr. Bull.* 21 356–360. 10.1177/156482650002100403

[B130] Upadhyaya H. D. Gowda C. L. L. Reddy V. G. (2007). Morphological diversity in finger millet germplasm introduced from Southern and Eastern Africa. *J. SAT Agric. Res.* 3 1–3.

[B131] Upadhyaya H. D. Ramesh S. Sharma S. Singh S. K. Varshney S. K. Sarma N. D. R. K. (2011). Genetic diversity for grain nutrients contents in a core collection of finger millet (*Eleusine coracana* (L.) Gaertn.)germplasm. *Field Crops Res.* 121 42–52. 10.1016/j.fcr.2010.11.017

[B132] Vadivoo A. S. Joseph R. Ganesan N. M. (1998). Genetic variability and diversity for protein and calcium contents in finger millet (*Eleusine coracana* (L.)Gaertn) in relation to grain color. *Plant Foods Hum. Nutr.* 52 353–364. 10.1023/A:1008074002390 10426122

[B133] Van Bel A. J. E. (1990). Xylem-phloem exchange via the rays: the undervalued route of transport. *J. Exp. Bot.* 41 631–644. 10.1093/jxb/41.6.631

[B134] Van Campen D. R. Glahn R. P. (1999). Micronutrient bioavailability techniques: accuracy, problems and limitations. *Field Crops Res.* 60 93–113. 10.1016/S0378-4290(98)00135-X

[B135] Varshney R. K. Hoisington D. A. Tyagi A. K. (2006). Advances in cereal genomics and applications in crop breeding. *Trends Biotechnol.* 24 490–499. 10.1016/j.tibtech.2006.08.006 16956681

[B136] Verslues P. E. Batelli G. Grillo S. Agius F. Kim Y. S. Zhu J. (2007). Interaction of SOS2 with nucleoside diphosphate kinase 2 and catalase reveals a point of connection between salt sress and H2O2 signaling in *arabidopsis thaliana*. *Mol. Cell. Biol.* 27 7771–7780. 10.1128/MCB.00429-07 17785451 PMC2169147

[B137] Véry A. A. Sentenac H. (2002). Cation channels in the *Arabidopsis* plasma membrane. *Trends Plant Sci.* 7 168–175. 10.1016/S1360-1385(02)02262-8 11950613

[B138] Ward J. M. Mäser P. Schroeder J. I. (2009). Plant ion channels: gene families, physiology, and functional genomics analyses. *Annu. Rev. Physiol*. 71 59–82. 10.1146/annurev.physiol.010908.163204 18842100 PMC4790454

[B139] Wang X. D. Harrington G. Patrick J. W. Oﬄer C. E. Fieuw S. (1995). Cellular pathway of photosynthate transport in coats of developing seed of *Viciafaba* L. and *Phaseolus vulgaris* L. II.Principal cellular site(s) of eﬄux. *J. Exp. Bot.* 46 49–63. 10.1093/jxb/46.1.49

[B140] Wang Y. J. Yu J. N. Chen T. Zhang Z. G. Hao Y. J. Zhang J. S. (2005). Functional analysis of a putative Ca^2+^ channel gene TaTPC1 from wheat. *J. Exp. Bot.* 56 3051–3060. 10.1093/jxb/eri302 16275671

[B141] Weaver C. M. Plawecki K. L. (1994). Dietary calcium: adequacy of a vegetarian diet. *Am. J. Clin. Nutr* 59 1238S–1241S. 8172128 10.1093/ajcn/59.5.1238S

[B142] Weaver C. M. Proulx W. R. Heaney R. (1999). Choices for achieving adequate dietary calcium with a vegetarian diet. *Am. J. Clin. Nutr.* 70 543s–548s. 10479229 10.1093/ajcn/70.3.543s

[B143] Welch R. M. Graham R. D. (1999). A new paradigm for world agriculture: meeting human needs: productive, sustainable nutritious. *Field Crops Res.* 60 1–10. 10.1016/S0378-4290(98)00129-4

[B144] Welch R. M. Graham R. D. (2002). Breeding crops for enhanced micronutrient content. *Plant Soil* 245 205–214. 10.1023/A:1020668100330

[B145] White P. J. Broadley M. R. (2003). Calcium in plants. *Ann. Bot.* 92 487–511. 10.1093/aob/mcg164 12933363 PMC4243668

[B146] White P. J. Broadley M. R. (2005). Biofortifying crops with essential mineral elements. *Trends Plant Sci.* 10 586–593. 10.1016/j.tplants.2005.10.001 16271501

[B147] Wolswinkel P. (1992). Transport of nutrients into developing seeds: a review of physiological mechanisms. *Seed Sci. Res.* 2 59–73. 10.1017/S096025850000115X

[B148] Wyatt S. E. Tsou P. L. Robertson D. (2002). Expression of the high capacity calcium binding domain of calreticulin increases bioavailable calcium stores in plants. *Transgenic Res.* 11 1–10. 10.1023/A:1013917701701 11874098

[B149] Yadav S. Gaur V. S. Jaiswal J. P. Kumar A. (2014). Simple sequence repeat (SSR) analysis in relation to calcium transport and signaling genes reveals transferability among grasses and a conserved behaviour within finger millet genotypes. *Plant Syst. Evol.* 300 1561–1568. 10.1007/s00606-014-0982-3

[B150] Yamanaka T. Nakagawa Y. Mori K. Nakano M. Imamura T. Kataoka H. (2010). MCA1 and MCA2 that mediate Ca^2+^ uptake have distinct and overlapping roles in *Arabidopsis*. *Plant Physiol*. 152 1284–1296. 10.1104/pp.109.147371 20097794 PMC2832256

[B151] Yi H. Juergens M. Jez J. M. (2012). Structure of soybean β-cyanoalanine synthase and the molecular basis for cyanide detoxification in plants. *Plant Cell* 24 2696–2706. 10.1105/tpc.112.098954 22739827 PMC3406924

[B152] Zhang B. Chen P. Shi A. Hou A. Ishibashi T. Wang D. (2009). Putative quantitative trait loci associated with calcium content in soybean seed. *J. Heredity* 100 263–269. 10.1093/jhered/esn096 18984858

[B153] Zhao Y. Mette M. F. Gowda M. Longin C. F. H. Reif J. C. (2014). Bridging the gap between marker-assisted and genomic selection of heading time and plant height in hybrid wheat. *Heredity* 112 638–645. 10.1038/hdy.2014.1 24518889 PMC4023446

[B154] Zhou J. R. Erdman J. W. (1995). Phytic acid in health and disease. *Crit. Rev. Food Sci. Nutr.* 35 495–508. 10.1080/10408399509527712 8777015

